# Pediatric-Inspired Regimens in the Treatment of Acute Lymphoblastic Leukemia in Adolescents and Young Adults: A Systematic Review

**DOI:** 10.3390/curroncol30090625

**Published:** 2023-09-20

**Authors:** Aida Zeckanovic, Philipp Fuchs, Philip Heesen, Nicole Bodmer, Maria Otth, Katrin Scheinemann

**Affiliations:** 1 Department of Oncology, University Children’s Hospital Zurich, 8032 Zurich, Switzerland; philipp.fuchs@kispi.uzh.ch (P.F.); nicole.bodmer@kispi.uzh.ch (N.B.); maria.otth@kispisg.ch (M.O.); 2 Children’s Research Center, University Children’s Hospital Zurich, 8032 Zurich, Switzerland; 3 Faculty of Medicine, University of Zurich, 8006 Zurich, Switzerland; philip.heesen@uzh.ch; 4 Division of Hematology/Oncology, Children’s Hospital of Eastern Switzerland, 9006 St. Gallen, Switzerland; katrin.scheinemann@kispisg.ch; 5 Faculty of Health Sciences and Medicine, University of Lucerne, 6000 Lucerne, Switzerland; 6 Department of Pediatrics, McMaster Children’s Hospital and McMaster University, Hamilton, ON L8S 4L8, Canada

**Keywords:** acute lymphoblastic leukemia, adolescents and young adult, protocol, pediatric-inspired, survival

## Abstract

Adolescents and young adults (AYA) with acute lymphoblastic leukemia (ALL) have significantly worse outcomes than their younger counterparts. Current treatment guidelines rely mostly on non-randomized retrospective studies. We performed a systematic review of studies published within the last 15 years comparing pediatric-inspired regimens (PIR) versus adult-type regimens or performing an age-stratified analysis of outcomes in the AYA population. Due to the heterogeneity of data, a meta-analysis was not possible. However, the gathered data show a trend toward improvement in outcomes and an acceptable toxicity profile in patients treated with PIRs compared to conventional adult-type regimens. There is still room for further improvement, as older patients within the AYA population tend to perform poorly with PIR or conventional adult-type chemotherapy. Further randomized studies are needed to develop an optimal treatment strategy for AYA with ALL.

## 1. Introduction

While the long-term prognosis of children with acute lymphoblastic leukemia (ALL) has improved in recent decades, the outcome of adolescents and young adults (AYA) patients aged from 15 to 39 years remains markedly worse than that of their younger counterparts [1]. Based on population-based data from the EUROCARE-5 study, which monitors the survival of cancer patients in Europe, the 5-year relative survival of children aged 0–14 years is 85.8%. In comparison, the relative survival rates in the adolescent (15–19 years) and young adult (20–39 years) age groups were 62.2% and 52.8%, respectively [2].

Due to the broad age range of the AYA population, these patients are treated in pediatric as well as adult settings with a myriad of different protocols. However, treating ALL in this age group is a challenge not only due to an increased incidence of unfavorable cytogenetic aberrations but also due to unique psychosocial circumstances as well as higher treatment-related toxicity compared to younger children [3]. Based on the current data and expert opinion guidelines, the best therapeutic approach for an AYA patient with ALL is to use a pediatric-inspired regimen (PIR) [3,4,5,6,7]. Compared to conventional adult-type protocols, PIRs tend to have more therapy elements and encompass higher cumulative doses of asparaginase, vincristine, and steroids, in addition to a generally longer maintenance phase [5]. The most prominent examples of PIR are protocols incorporating a Dana–Faber Cancer Institute (DFCI) or Berlin–Frankfurt–Münster (BFM) study group backbone. However, data on treatment strategies in AYA and ALL are limited due to the lack of randomized comparative studies and thus prone to bias, making interpretation and comparison difficult.

The purpose of this systematic review is to provide a comprehensive synthesis of published comparative studies examining the outcomes and toxicity of AYA patients treated for ALL with PIR versus conventional adult regimens. Furthermore, we would like to summarize the available data on age-stratified outcomes and adverse events (AE) in AYA patients receiving PIR treatment.

## 2. Materials and Methods

We conducted the systematic literature search according to the PRISMA guidelines in PubMed in November 2022 [8]. The search strategy was built around the following three concepts: “acute lymphoblastic leukemia”, “adolescents and young adults”, and “treatment protocol/strategy” (Supplementary Data). Publications on myeloid leukemia and those with animal models were omitted through the search strategy. We restricted the search to studies published between November 2007 and November 2022. The inclusion criteria were given through the PICO framework [8]. The population included AYA cancer patients diagnosed with ALL. The AYA population was defined as patients diagnosed between the ages of 15 and 39 years, or at least 75% of the study population had to be within this range. The intervention corresponded to the treatment protocol, either a pediatric, pediatric-inspired, or adult protocol. Depending on the data provided in the eligible publications, we aimed to compare either adult-type versus pediatric/pediatric-inspired protocols or pediatric/pediatric-inspired protocols stratified by different age categories. The envisaged outcomes included survival (e.g., overall (OS) or event-free survival (EFS)), toxicity (e.g., toxic death, admission to the ICU), or reasons for the protocols used. However, the final reporting of these outcomes depended on whether the data were provided in the eligible publications or not. 

Two authors performed the title and abstract screening (MO, AZ) and full-text screening (AZ, PF) each. Discrepancies between reviewers one and two were solved by a third reviewer (KS) using the same criteria. We extracted the data from the eligible studies onto a standard sheet, including the first author, year of publication, study design, patients’ characteristics, information on the treatment protocol, and the outcomes assessed. We assessed the quality, relevance, and reliability of each included study by using the appropriate critical appraisal tool from the Joanna Briggs Institute (JBI) (https://jbi.global/critical-appraisal-tools) (accessed on 15 October 2022), including the checklists for cohort studies. Since the tools from the Joanna Briggs Institute do not have predefined categorizations, we defined a classification with three categories. If all criteria of the respective checklist were fulfilled, we assigned the study “Quality 1”. If one or two criteria were not fulfilled, we assigned the study to “Quality 2”. If three or more criteria were not fulfilled, the study was assigned “Quality 3”.

The protocol for this review was published on Prospero (https://www.crd.york.ac.uk/prospero; ID: CRD42022384667) (accessed on 27 December 2022).

## 3. Results

### 3.1. Description of Studies and Regimens

**Figure 1:**
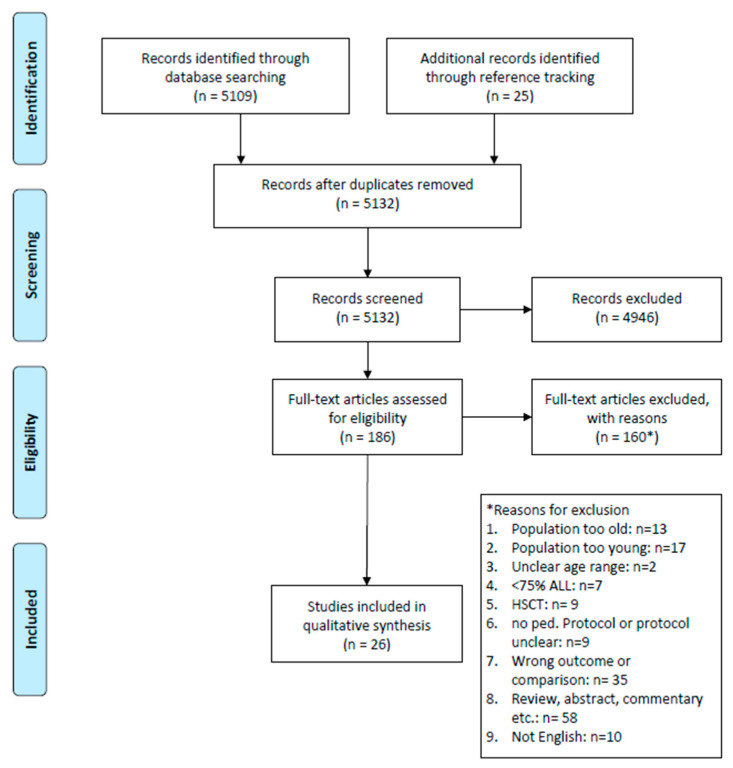
PRISMA flow diagram.

The literature search identified 5132 publications. A total of 168 potentially relevant full-text articles were retrieved for further evaluation. Among these, 26 met the inclusion criteria for our systematic review (Figure 1, Table 1, Supplementary Data) [9,10,11,12,13,14,15,16,17,18,19,20,21,22,23,24,25,26,27,28,29,30,31,32,33,34]. Fifteen of the included studies (57%) had a prospective design [9,14,16,19,20,22,23,25,26,27,28,29,30,32,33]. 

Twelve studies comparing the outcomes and/or toxicity of PIR vs. conventional adult-type regimens are summarized in Table 2 [10,11,12,15,17,18,21,22,24,29,30,31]. None of the studies were randomized controlled trials. Four studies included patients given PIR that were compared with historical controls receiving conventional adult regimens [12,24,29,30]. Others compared patients treated with PIR and adult protocols during approximately the same time periods. One study did not use any risk-adapted treatment for PIR; the others used some sort of risk stratification of patients [18]. 

Five studies had a median follow-up shorter than two years for at least one analyzed group [12,15,17,18,31], and one study did not specify the duration of follow-up [21]. Additionally, two studies had significantly longer follow-ups for the patients treated with conventional adult protocols [12,24], whereas another had significantly longer follow-ups for the patients treated with PIR [15]. In most studies, the compared groups had well-matched age distributions. However, in two studies, the group receiving the conventional adult treatment was slightly older than the PIR group [21,31], whereas the opposite was true for one study [12].

Nineteen studies, summarized in Table 3, describe the treatment outcomes or toxicities in different age groups, either within the defined AYA range or as a comparison to younger or older patients. Three studies have a shorter follow-up than 2 years [12,17,19], while one has a significantly shorter follow-up for the oldest analyzed age group [32]. Two studies contain data gathered during two different periods [9,12].

The regimens of both PIR and conventional adult protocols were different between the studies. The dosing regimens are described in detail in the corresponding articles. However, in general, PIR had higher cumulative dosages of chemotherapeutic agents such as corticosteroids, vincristine, and methotrexate and incorporated more asparaginase. Regarding the studied populations, the type of ALL (B-ALL, T-ALL, or BCR–ABL positive ALL) differed between the studies but was consistent within each study (Table 1). Quality appraisal according to the JBI quality assessment scale for cohort studies is shown in Table 1.

### 3.2. Treatment Outcomes and Toxicity in AYA Patients When Treated with PIR versus Conventional Adult Regimens

A statistically significant improvement in OS in patients given PIR compared to conventional adult protocols was reported in 6 out of 11 studies (Table 2) [10,11,12,17,21,22]. Even in the five studies that found that OS did not statistically significantly differ between the two types of treatment strategies, the reported OS for PIR tended to be higher than in the adult-type regimens [18,24,29,30,31].

The limited data and their accuracy did not allow us to perform a meta-analysis to assess the impact of the treatment strategy used. Even the consultation with the guidance of Tierney JF et al. did not allow a calculation of the hazard ratios (HR) [35]. Only three studies report HRs, but the HRs were given for different time points (2 years, 3 years, and 5 years), which further impeded performing a meta-analysis [17,21,31]. 

The clinical endpoints other than OS were very heterogeneous among the studies (Table 2). Nevertheless, a similar trend can be seen with the reported relapse rates, event-free survival (EFS), relapse-free survival (RFS), and disease-free survival (DFS), which were described in 10/12 studies. Three studies demonstrate an improvement in EFS, two in RFS, and two in DFS for the entire analyzed group [10,11,12,18,21,22,24,31]. Additionally, Cheng et al. report a significant improvement in 5-year EFS for a sub-group of untransplanted patients, and Ganesan et al. report, in addition to the improvement in the relapse rate and RFS, a trend toward improvement of EFS (*p* = 0.054) in the analyzed ALL patients [15,18]. 

Altogether, nine out of twelve studies report an improvement in either OS or EFS/RFS/relapse rate or both, while three studies found equivalent results. No study reported statistically significant superior outcomes with conventional adult-type chemotherapy.

The results are less impressive for post-induction complete remission rate (CR), reported by nine studies, with only two showing a statistically significant increase in CR rate in patients given PIR compared to patients given conventional adult protocols (Table 2) [10,11,12,18,22,24,29,30,31].

Regarding toxicity, the studies show increased toxicity with PIR compared to conventional adult protocols. Most commonly, an increased incidence of pancreatitis, hypofibrinogenemia, neuropathy, hepatic toxicity, and infections was reported by the studies [10,22,24,29] (Table 2). However, most toxicities were described as mild and manageable with supportive care [10,24]. Most importantly, except for one study from India, no other studies reported significantly increased induction-related mortality (IRM) or treatment-related deaths [15,17,24,29,31]. Almanza-Huante et al. report a decrease in IRM and TRD with a modified pediatric protocol [12].

### 3.3. Age-Stratified Analysis of Outcomes and Toxicities in AYA Patients Treated with PIR

Nineteen studies were included in this section. These studies examine outcomes between different age groups of AYA ALL patients treated with PIR (Table 1 and Table 3). For two studies, an age-stratified PIR vs. adult-type protocol comparison is available. Almanza-Huante et al. found a significant increase in CR at the end of induction and OS in patients aged 21–43 years treated with PIR as opposed to an adult-type protocol, with no increase in IRM. However, the follow-up duration was significantly shorter in patients who were treated with PIR, which might impact the reported OS. Conversely, Rytting et al. found no difference in OS between patients aged ≥21 and <21 years treated with PIR versus an adult-type protocol.

The remaining studies examined age-stratified outcomes or toxicity in patients treated exclusively with PIR (Table 3). Six of these studies report a significantly superior OS in younger age groups [13,14,23,25,27], with two additional studies also showing a trend towards inferior OS with increasing age (*p* = 0.057 and *p* = 0.055) [17,30]. On the contrary, four studies found no significant difference in OS between different age groups [16,19,20,28]. Similarly, heterogeneous results can be found for EFS, with four studies showing better outcomes for younger age groups [14,23,25,32] and five studies showing no significant difference between the age groups [15,16,17,27,28]. However, the studies identifying age as a significant predictor of EFS belong to those with the largest number of enrolled patients and thus the largest statistical power [14,23,25,32].

Furthermore, there are several adverse events (AE) whose incidence seems to be increasing with age (Table 3). Hough et al. report an overall increased cumulative incidence of AEs for patients aged >10 years and a significantly shorter time to the first AE after the start of treatment [23]. The AEs most commonly reported with increasing age are thrombosis, hypofibrinogenemia, hepatic injury, and infectious complications [9,14,26,28,30,32,33]. The risk of ICU admission and IRM does not seem to increase with age [14,32,33]. However, two studies report an increased incidence of toxic deaths in remission in older age groups [14,23]. Several studies show that the incidence of avascular osteonecrosis (ON) reaches its peak in the AYA age group, with a decrease in frequency in younger children and adults [32,33,34]. Furthermore, Valtis et al. show an increased risk for ON with the use of pegylated asparaginase, which is used with increasing frequency in new generations of PIR [34].

## 4. Discussion

This systematic review of 26 published comparative studies reporting outcomes of AYA patients with ALL shows a trend towards improvement in outcomes and an acceptable toxicity profile in patients treated with PIRs, compared to conventional adult-type regimens. While direct comparison and analysis were difficult due to heterogeneous study populations, treatment settings, treatment eras, and treatment protocols, most of the included studies nevertheless reported an increase in survival with the use of PIR.

Despite PIRs quickly becoming the standard of care for ALL treatment in the AYA population, further improvements are necessary. Our systematic review demonstrates a clear trend towards poorer survival with increasing age, even when using PIRs [13,14,23,25,27]. This is most likely due to a combination of higher therapy-related toxicity, requiring dose reductions and protocol adjustments and causing treatment delays, as well as disease biology [14,23]. 

Since PIRs are expected to be more intensive than adult-type regimens, an increase in treatment-related toxicity and adverse events is expected. However, our data show that while certain AEs increase with age, their toxicity is mostly manageable. Furthermore, PIRs also showed good results even in lower- and middle-income countries [10,12,17,18,19,31]. Yet, setting the age limit for the feasibility of these protocols is crucial so that the added toxicity and mortality do not surpass the positive effect of PIR on survival. With our analysis of age-stratified outcomes, we were unable to identify the optimal upper age limit for PIR.

Some of the included studies report low completion rates, high treatment abandonment rates, and large proportions of patients requiring dose reductions and treatment delays with increasing age [9,19,22,28]. In the study by Ribera et al., there were significantly more delays during reinductions and dose modifications for vincristine or asparaginase in young adults than in adolescents (*p* = 0.04 and *p* = 0.03, respectively). Adjustments to the protocol or alterations in the treatment strategy are more likely if the physician is unfamiliar with the protocol [9]. This is highlighted by the study by Gupta et al., which found a trend towards inferior EFS in patients treated with PIR in adult centers versus pediatric centers (HR 1.92, 95% CI 0.99–3.75, *p* = 0.06). The magnitude of the disparity between the two types of treatment centers persisted over time and even after adjusting for sociodemographic factors. This may be partially explained by a larger proportion of AYA patients treated in pediatric centers being registered on clinical trials (86/123 (69.9%) vs. 7/152 (4.6%), *p* < 0.001) or by better psychosocial support [21].

However, it is also well documented that treatment completion in the AYA age group is often low despite the physicians’ familiarity with the protocol. In the study by Advani et al., 57% of the AYA patients completed all therapy according to the COG AALL0232 PIR protocol versus 74% of the patients below 18 years of age [9]. Furthermore, Hayakawa et al. also report frequent terminations due to AEs or patients’ wishes. The latter happened predominantly during maintenance therapy [22]. This is presumably due to long, arduous PIR treatment programs resulting in low motivation. An alternative explanation for treatment termination in some low- and middle-income countries is socioeconomic factors, such as needing to pay for treatment out of pocket [19]. 

All these factors make translating the conclusions of this systematic review into clinical practice a precarious endeavor. The main limitations of our systematic review are based on the limited available data, including the lack of randomized studies and the heterogeneity in reporting the outcomes. 

Randomized studies are needed to establish international treatment standards for AYA patients with ALL, improve risk stratification, and evaluate treatment response assessment using minimal disease measurements. Such studies would also ensure better data collection, adherence to the treatment dosing and schedule, integrated management of the most common AEs, and better support for physicians unfamiliar with the pediatric-inspired treatment protocols. Without them, we may not be able to definitively elucidate the magnitude of the influence of various treatment elements on improved outcomes (the prescribed regimen, locus of care, physicians´ experience with the protocol, compliance, socioeconomic and psychosocial factors, etc.).

## 5. Conclusions

Unfortunately, the gathered data do not allow for clear conclusions about the best treatment protocols to use in the AYA population. The trend towards improved outcomes with PIR must be viewed with caution, as non-randomized trials are prone to bias and difficult to compare and interpret. We should strive to enroll AYAs with ALL in randomized controlled trials of PIR vs. conventional adult-type protocols to definitively elucidate the best treatment strategy.

## Figures and Tables

**Table 1:** Characteristics of the studies included in this review.

**Author, Year, Country**	**Years of Recruitment**	**Diagnosis**	**PIR**	**N of Pediatric** **Patients (%)**	**Adult Protocol**	**N of Adult** **Patients (%)**	**Median Age (Range)** **in Years**	**JBI Score**
**Studies comparing adolescents and young adult patients receiving a PIR or adult regimen**								
Al-Khabori, 2010 Canada [11]	January 1990–March 2007	T-ALL	DFCI regimen	32 (44)	9203ALL, Protocol C, Hyper-CVAD, MRC UKALL XII/ECOG E2993	40 (56)	30.8 (17–69)	1
Alacacioglu, 2014 Turkey [10]	March 2006–October 2012	ALL	BFM-Like	20 (40)	Hyper-CVAD	30 (60)	Overall: 27.5 (18–59) PIR: 25 Hyper-CVAD: 30.5	2
Almanza-Huante, 2021 Mexico [12]	PIR: March 2016– June 2019 hyper-CVAD: February 2009–June 2015	BCR–ABL negative ALL	modified ALL-BFM 90, modified CALGB C10403	73 (30)	Hyper-CVAD	173 (70)	Overall: 22 (14–43) PIR: 24 Hyper-CVAD: 20	1
Cheng, 2022 Taiwan [15]	2008–2019	VHR-ALL	TPOG-ALL-2002 protocol	16 (59)	Hyper-CVAD/HD-Methotrexate and Cytarabine	11 (41)	PIR: 24.3 (18–36) Hyper-CVAD: 33 (20–40)	1
Ganesan 2021 India [17]	2012–2017	ALL (including MPAL)	MCP-841, BFM-90, -95 or -2000, COG Protocols	1002 (88)	GMALL, Hyper-CVAD, UKALL	139 (12)	Overall: range 15–29 PIR: 20 Adult: 23	1
Ganesan, 2018 India [18]	January 2000–December 2014	BCR–ABL negative ALL	BFM 95, SR arm	147 (63)	MCP-841 (22%) GMALL (9%) INCTR (4%) UKALL (2%)	85 (37)	Overall: 21 (18–30) PIR: 21.8 Adult: 22.4	2
Gupta, 2019, Canada [21]	1992–2011	ALL	DFCI Protocol 91-01	123 (54)	Not specified	106 (46)	Range 15–21 Pediatric centers: mean 16 ± 1 Adult centers: mean 19 ± 1	1
Hayakawa, 2014, Japan [22]	August 2002–October 2009	BCR–ABL negative B-ALL	ALL202	139 (57)	ALL97-U	104 (43)	19 (15–24)	1
Tantiworawit, 2019, Thailand [31]	January 2007–December 2017	ALL	TPOG protocol	35 (33)	Hyper-CVAD or GMALL protocol	75 (67)	Overall: 26 (15–63) Adult: 29.5 (16–63) PIR: 24 (15–39)	2
Rytting, 2014 USA [30]	October 2006–April 2012	BCR–ABL negative ALL	Augmented BFM regimen	85	Historic Hyper-CVAD cohort	71	PIR: 21 (13–39) Adult: 26 (16–40)	1
Rytting, 2016, USA [29]	October 2006–March 2014	BCR–ABL negative ALL	Augmented BFM regimen	106	Historic Hyper-CVAD cohort	102	PIR: 22 (13–39) Adult: 27 (15–40)	1
Kliman, 2017, Canada [24]	PIR: February 2008–November 2014 Adult: February 2003–July 2008	SR BCR–ABL negative ALL	Modification of DFCI 01–175	22	Comparative adult ALL protocols (not exactly specified)	25	Overall: 24.5 (18–40) PIR: 27.6 Adult: 23.5	2
**Studies comparing age-stratified outcomes and toxicity in adolescents and young adult patients receiving a PIR or adult regimen**								
**Ref.**	**Years of recruitment**	**Diagnosis**	**Protocol**	**Age groups and N of patients**				**JBI score**
Advani, 2021, USA [9]	CALGB 10403: 2007–2012 COG AALL0232: 2004–2011	B- or T-precursor ALL	PIR: CALGB 10403 and COG AALL0232 (arm identical to CALGB 10403)	CALGB 10403: - 16–21 years: *n* = 94 (33%) - 22–30 years: *n* = 131 (45%) - 31–39 years: *n* = 64 (22%) COG AALL0232: - 16–21 years: *n* = 146 (92%) - 22–30 years: *n* = 12 (8%)				1
Almanza-Huante, 2021 Mexico [12]	PIR: March 2016– June 2019 Adult: February 2009–June 2015	BCR–ABL negative ALL	PIR: modified ALL-BFM 90, modified CALGB C10403 Adult: Hyper-CVAD	14–20 years: - PIR: *n* = 23; Hyper-CVAD: *n* = 69 21–43 years: - PIR *n* = 50; Hyper-CVAD *n* = 68				1
Brandwein, 2014 Canada [13]	June 2000–June 2011	BCR–ABL negative ALL	PIR: DFCI 91-01	17–<34 years: *n* = 73 (47%) 34–<50 years: *n* = 54 (35%) 50–60 years: *n* = 29 (19%)				1
Burke, 2022, USA [14]	January 2004–January 2011	HR B-ALL, excluding DS patients	PIR: COG AALL0232	<16 years: *n* = 2443 (=younger children) 16–30 years: *n* = 597 (=AYA population)				1
Cheng, 2022, Taiwan [15]	2008–2019	VHR-ALL	PIR: TPOG-ALL-2002 protocol	18–25 years: *n* = 7 26–34 years: *n* = 9				1
De Angelo, 2015, USA and Canada [16]	August 2002–February 2008	ALL (excl. mature B-cell ALL)	PIR: DFCI Pediatric ALL Consortium regimen/DFCI Adult: ALL Consortium Protocol 01–175	18–29 years: *n* = 48 (52%) 30–50 years: *n* = 44 (48%)				2
Ganesan, 2021, India [17]	2012–2017	ALL (including MPAL)	PIR: MCP-841^7^, BFM-90, -95 or -2000), COG Adult: GMALL, Hyper-CVAD, UKALL	15–17 years: *n* = 403 (29.1%) 18–24 years: *n* = 688 (49.7%) 25–29 years: *n* = 292 (21.2%)				1
Gómez-De León, 2022, Mexico [19]	2016–2020	BCR–ABL negative B-ALL	PIR: Modified BFM protocol	16–19 years: *n* = 31 20–29 years: *n* = 33 30–39 years: *n* = 16 ≥40 years: *n* = 11				2
Greenwood, 2021, Australia [20]	July 2012–June 2018	ALL	PIR: ANZCHOG Study 8 protocol	Median 22.7 years (16–38 years), analyzed as a continuous variable				1
Hough, 2016 UK, Ireland [23]	October 2003–June 2011	BCR–ABL negative B-ALL	PIR: UKALL2003	16–24 years: *n* = 2287 10–15 years: *n* = 610 <10 years: *n* = 229				1
Valtis, 2022, USA [34]	2000–2018	ALL	PIR: DFCI ALL Consortium 00-001, 05-001, 01-175, 06-254	15–19 years: *n* = 138 (38%) 20–29 years: *n* = 110 (30%) 30–39 years: *n* = 62 (17%) 40–50 years: *n* = 57 (16%)				1
Toft, 2018, Denmark, Estonia, Finland, Iceland, Lithuania, Norway, Sweden [32]	July 2008–December 2014	T-ALL or BCR–ABL negative B-ALL, excluding patients with DS	PIR: NOPHO ALL2008 protocol	1–9 years: *n* = 1022 (68%) 10–17 years: *n* = 266 (18%) 18–45 years: *n* = 221 (14%)				1
Toft, 2016, Countries see above [33]	July 2008–April 2013	T-ALL or BCR–ABL negative B-ALL, excluding patients with DS	PIR: NOPHO ALL2008 protocol	1–9.9 years: *n* = 733 (69%) 10–14 years: *n* = 118 (11%) 15–17 years: *n* = 77 (7%) 18–26 years: *n* = 70 (7%) 27–45 years: *n* = 64 (6%)				1
Rytting, 2014, USA [30]	October 2006–April 2012	BCR–ABL negative ALL	PIR: Augmented BFM regimen	12–21 years: *n* = 44 (52%) 22–40 years: *n* = 41 (48%)				1
Rytting, 2016, USA [29]	October 2006–March 2014	BCR–ABL negative ALL	PIR: Augmented BFM regimen Adult: Historical cohort (treated with Hyper-CVAD	13–21 years: - PIR *n* = 99 (50%); adult *n* = 100 (50%) 22–40 years: - PIR *n* = 53; adult *n* = 81				1
Ribera, 2008, Spain [28]	June 1996–June 2005	SR ALL	PIR: PETHEMA ALL-96 protocol	15–18 years: *n* = 35 (43%) 19–30 years: *n* = 46 (57%)				1
Ribera, 2020, Spain [27]	August 2008–April 2018	SR BCR–ABL negative ALL	PIR: PETHEMA ALLRE08	15–18 years: *n* = 38 (43%) 19–30 years: *n* = 51 (57%)				1–2
Quist-Paulsen, 2020, Countries as Toft et al. [25]	July 2008–March 2016	T-ALL	PIR: NOPHO ALL2008 protocol	1–9: *n* = 117 (42%) 10–17: *n* = 78 (28%) 18–45: *n* = 83 (30%)				1
Rank, 2018, Countries same as Toft et al. [26]	July 2008–February 2016	BCR–ABL negative ALL	PIR: NOPHO ALL2008 protocol	1–9.9 years: *n* = 1192 (67%) 10–17.9 years: *n* = 306 (17%) 18–45 years: *n* = 274 (16%)				1

ALL: Acute lymphoblastic leukemia; DFCI: Dana–Faber Cancer Institute; Hyper-CVAD: Hyperfractionated cyclophosphamide, vincristine, doxorubicin, dexamethasone; BFM: Berlin–Frankfurt–Münster Study Group; CALGB: Cancer and Leukemia Group B Study Group; MPAL: Mixed phenotype acute leukemia; SR: Standard risk; HR: High risk; VHR: Very high risk; BCR–ABL positive ALL: ALL with BCR–ABL translocation; TPOG: Taiwan Pediatric Oncology Group; MCP-841: Multicentre protocol 841; COG: Children’s Oncology Group; GMALL: German Multicentre ALL Protocol; INCTR: International Network for Cancer Treatment and Research; DS: Down syndrome; ANZCHOG: Australian and New Zealand Children’s Haematology/Oncology Group; NOPHO: Nordic Society of Paediatric Haematology and Oncology; PETHEMA: Programa Español de Tratamientos en Hematología.

**Table 2:** Main findings of studies comparing outcomes and toxicity in adolescents and young adult patients receiving a pediatric/pediatric-inspired (PIR) or adult regimen.

Ref.	CR Rate after Induction (PIR vs. Adult)	EFS/RFS/DFS/Relapse Rate (PIR vs. Adult)	OS (PIR vs. Adult)	Toxicity (PIR vs. Adult)	The Median Duration of Follow-Up in Months (Range)
AL-Khabouri, 2010 [11]	84% vs. 93%	3-year RFS: 89% vs. 24%; *p* < 0.0001	3-year OS: 81% vs. 44%; *p* = 0.0003 5-year OS: 75% (85% CI: 55–88%) vs. 25% (95% CI:13–39%); *p* = 0.0003	NA	54 (13–238)
Alacacioglu, 2014 [10]	95% vs. 96%	Mean RFS: 53.9 ± 5.4 vs. 39.1 ± 6.8 months; *p* = 0.009	Mean OS: 55.1 ± 4.9 vs. 41.5 ± 6.4 months; *p* = 0.012 5-year OS: 59% vs. 34%	No anaphylactic reactions to *E. coli* L-ASP, no pancreatitis, or venous complications. Mild elevation of liver enzymes. No complications caused a delay in either protocol.	37
Almanza-Huante, 2021 [12]	79.5% vs. 64.2%; *p* = 0.02	Relapse rate: 44.1% vs. 60%; *p* = 0.04	OS: 18.5 [95% CI, 13.61–23.43] vs. 11.08 months [95% CI, 7.33–14.83]) 2-year OS: 41.5% vs. 28.1%; *p* = 0.01	IRM: 1.4% PIR vs. 8% hyper-CVAD (*p* = 0.04) TRD due to infection: 3.3% PIR vs. 28.1% hyper-CVAD	Hyper-CVAD: 101 BFM: 32 CALGB: 22
Cheng, 2022 [15]	NA	5-year EFS: 71.6 ± 12.2% vs. 45.5 ± 15.0%; *p* = 0.152 HR: 0.42; *p* = 0.16 5-year EFS (untransplanted patients): 83.3% ± 10.8% vs. 28.6% ± 17.1%; *p* = 0.039 HR 4.19, *p* < 0.05	NA	Toxic death: *n* = 1 in both groups	PIR: 60 months (6–108) Adult: 20 months (2–127)
Ganesan, 2021 [17]	NA	2-year EFS: 56.6% vs. 52.1%; *p* = 0.730 HR with 95% CI: 1.05 (0.81–1.35); *p* = 0.736 2-year RFS: 75.1% vs. 75.4%; *p* = 0.702	2-year OS: 75.4% vs. 59.0%; *p* < 0.001 HR with 95% CI: 1.72 (1.29–2.29; *p* < 0.001 (univariate) 3.19 (1.95–5.22); *p* < 0.001 (multivariate)	NA	23 months (95% CI 6–38)
Ganesan, 2018 [18]	84% vs. 82%	5-year RFS: 51% vs. 35%; *p* = 0.027 5-year EFS: 40% vs. 27% *p* = 0.054	5-year OS: 43% vs. 33%; *p* = 0.2	IRM: 10% vs. 1% *p* = 0.001; major causes: sepsis, L-ASP associated thrombotic complications TRD: 12% vs. 2%; *p* = 0.031	21 months (0.3–165)
Gupta, 2019 [21]	NA	5-year EFS, treated between 2006 and 2011: pediatric center AYA 80.8 ± 5.8% vs. adult center with PIR 71.8% ± 7.2% vs. adult centers with adult protocols 60.0% ± 11.0%; *p* = 0.02	5-year OS, treated between 2006 and 2011: pediatric center 90.9 ± 4.3%, adult center with PIR 76.9 ± 6.82%, adult centers with adult protocols 65.0 ± 10.7.0%; *p* = 0.004	NA	NA
Hayakawa, 2014 [22]	94% (95% CI 88–97%) vs. 84% (75–90%)	5-year DFS: 67% (95% CI 58–75%) vs. 44% (33–55%), not statistically significant, but the *p*-value has not been shown	5-year OS: 73% (95% CI 64–80%) vs. 45% (35–55%) not statistically significant, but the *p*-value has not been shown	Sepsis, hepatic toxicity, and neuropathy were more frequent in PIR. No toxic deaths occurred during post-remission therapy due to severe adverse events.	PIR: 61 Adult: 67
Tantiworawit, 2019 [31]	88.2% vs. 79.2%, *p* = 0.23	2-year DFS: 47.1% vs. 24.7% (HR 1.73, 1.22–3.03, *p* = 0.04) Relapse rate: 34.3% vs. 54.2%, *p* < 0.01 DFS for BCR–ABL negative ALL: 46.8% vs. 18.7% (HR 2.16, 1.16–4.01, *p* = 0.01)	2-year OS 50.8% vs. 31.2% (HR 1.52, 0.83–2.78, *p* = 0.16) For BCR–ABL negative ALL 2-year OS of 59.4% vs. 31.8% (HR 2.03, 1.04–3.96, *p* = 0.03)	IRM: 2.9 vs. 5.6%, *p* = 0.53	11.6 (1–120)
Rytting, 2014 [30]	94% vs. 99%, *p* = 0.14	NA	3-year OS rate: 74% vs. 71%, not statistically significant, but the *p*-value has not been shown	See Table 3	40 (4–75)
Rytting, 2016 [29]	93% vs. 98%, *p*-value not shown	NA	5-year OS: 60% vs. 60%	Toxicity (PIR vs. adult): No significant difference for allergic reactions, liver enzyme and bilirubin elevation, ON, thrombosis, stroke-like events, neuropathy, bleeding, or deaths in CR Hypofibrinogenemia 35% vs.14%, *p* < 0.001 Pancreatitis 11% vs. 3%, *p* = 0.02 Induction infections grade 3–4: 22% vs. 45%, *p* < 0.001 Infections in CR in the first 60 days: 30% vs. 60%, *p* < 0.001	PIR: 66 months (17–107) Adult: 88 (1–152)
Kliman, 2017 [24]	100% vs. 86%, *p* = 0.095	3-year EFS: 80% vs. 45%, *p* = 0.019	3-year OS: 80% vs. 59%, *p* = 0.12	There were no significant differences between the incidence of candidemia, severe infection, thrombosis, pancreatitis, or toxic death.	Overall: 40.1 PIR: 36.8 Adult: 73.1

CR: Complete remission; EFS: Event-free survival; RFS: Relapse-free survival; DFS: Disease-free survival; OS: Overall survival; L-ASP: L-Asparaginase; Hyper-CVAD: Hyperfractionated cyclophosphamide, vincristine, doxorubicin, dexamethasone; BFM: Berlin–Frankfurt–Münster Study Group; NA: Not applicable; CI: Confidence interval; HR: Hazard ratio; AYA: Adolescents and young adults; TRD: Treatment-related deaths; IRM: Induction-related mortality.

**Table 3:** Main results of studies comparing age-stratified outcomes and toxicity in adolescents and young adult patients receiving a pediatric-inspired (PIR) or adult regimen.

Ref.	CR Rate after Induction	EFS/RFS/Relapse Rate	OS	Toxicity	Median Follow-Up in Months (Range)
Advani, 2021 [9]	NA	NA	NA	IRM with CALGB 10403 and COG AALL0232: 3.1% and 1.3%. Main Grade 3 and 4 toxicities with an incidence > 15%: hyperglycemia, bilirubin and ALT increases, febrile neutropenia, and infection. Post-induction mortality with CALGB 10403 and COG AALL0232: 1.3% and 0.8%. Main Grade 3 and 4 post-induction toxicities with an incidence > 15%: febrile neutropenia, infection, sensory neuropathy, hyperglycemia, bilirubin, AST and ALT increases, anaphylaxis. Increased age correlated with a decreased fibrinogen level and ALT increase in induction (OR 1.103; *p* = 0.0001 and OR 1.111; *p* = 0.0002) and post-induction therapy (OR 1.037; *p* = 0.039 and OR 1.045; *p* = 0.011).	NA
Almanza-Huante, 2021 [12]	Age group 14–20 years 71.0% PIR vs. 69.6% hyper-CVAD; *p* = 1.0	NA	Median OS 27.4 months (95% CI 9.5–45.3) in PIR vs. 15.4 months (8.5–22.3) in hyper-CVAD (*p* = 0.30)	IRM: 0% PIR vs. 10.1% hyper-CVAD (*p* = 0.18)	Hyper-CVAD: *n*= 101 BFM: *n* = 32 CALGB. N = 22
	Age group 21–43 years 84% PIR vs. 57.4% hyper-CVAD *p* = 0.02	NA	Median OS 16.9 months (95% CI 13.1–20.6) PIR vs. 9.2 months (95% CI 6–12.5) in hyper-CVAD (*p* < 0.01)	IRM: 2% PIR vs. 6.9% hyper-CVAD and 2% (*p* = 0.39)	
Brandwein, 2014 [13]	17–<34 years: 99% 34–50 years: 87% 50–60 years: 26% (*p* = 0.02)	NA	5-year OS (95% CI), univariate: 17–<34 years: 80% (67–88%) 34–50 years: 50% (35–63%) 50–60 years: 62% (42–77%) *p* = 0.001 Age (cont. variable) as a predictor of OS (*p* = 0.0046)		42 months (range 0.3–135 months)
Burke, 2022 [14]	NA	5-year EFS: 65.4 ± 2.2% for AYA vs. 78.1 ± 0.9% for younger patients (*p* < 0.0001) Age as a significant predictor of EFS as categorical (<16 vs., >16) and continuous variable in univariate and multivariable analysis (categorical univariate: *p* < 0.0001; continuious univariate: *p* < 0.0001; categorical multivariable: *p* = 0.018; continuous multivariable: *p* < 0.0001 respectively)	5-year OS: 77.4 ± 2.0% for AYA vs. 87.3 ± 0.7% for younger patients (*p* < 0.0001)	IRM 2.2% in AYA versus 1.6% in younger patients (*p* = 0.366) Toxicity Grade ≥ 3 in induction (AYA vs. younger): Hyperglycemia: 23.6% vs. 15.4% (*p* < 0.0001) Hyperbilirubinemia: 6.9% vs. 3.7% (*p* = 0.0007) Febrile neutropenia: 7.4% vs. 13.8% (*p* < 0.0001) There was no significant difference in thrombosis or pancreatitis. Toxicity Grade ≥ 3 in post-induction (AYA vs. younger): Mucositis: 18.2% vs. 11.7% (*p* = 0.0002) Peripheral neuropathy: 12.1% vs.7.8% (*p* = 0.001) Febrile neutropenia: 45.2% vs. 56.8% (*p* < 0.0001) Hyperbilirubinemia: 17.3% vs. 9.5% (*p* < 0.0001) Hepatic failure: 1.3% vs. 0.3% (*p* = 0.009) Deaths in remission: 5.7% vs. 2.4% (*p* < 0.0001), mostly Grade 5 infections.	NA
Cheng, 2022 [15]	NA	5-year EFS: 64.3 ± 21.0% for 18–25 years vs. 76.2 ± 14.8% for older group; *p* = 0.265	NA	NA	60 months (6–108)
DeAngelo, 2015 [16]	NA	4-year EFS (95% CI) age 18–29 vs. 30–50: 55% (39–69%) vs. 61% (44–74%), *p* = 0.61	4-year OS (95% CI) age 18–29 vs. 30–50: 68% (52–80%) vs. 65% (49–77%), *p* = 0.93	NA	54 (95% CI 49–60)
Ganesan, 2021 [17]	NA	2-year EFS, HR (95% CI) 15–17 years: 56.7%, ref. 18–24 years: 55.9%, 1.01 (0.83–1.23), *p* = 0.937 25–29 years: 55.4% 1.02 (0.80–1.30); *p* = 0.862 2-year RFS: 15–17 years: 74.8% 18–24 years: 75.3% 25–29 years: 75.4% *p* = 0.948 (log-rank)	2-year OS; HR (95% CI) 15–17 years: 76.6%, ref. 18–24 years: 73.0%; 1.20 (0.91–1.58), *p* = 0.203 25–29 years: 69.3%; 1.37 (0.99–1.89); *p* = 0.057	NA	23 (95% CI 6–38)
Gomez, 2022 [19]	NA	≥40 years with lower EFS: 8.3 months (95% CI 0–21.2; *p* = 0.006); no difference in the AYA groups Age as continuous variable HR (95% CI): 1.93 (0.99–1.07)	There was no statistically significant difference in OS between age groups. Age as continuous variable HR (95% CI): 1.03 (0.9–1.07)	Induction deaths: bleeding (*n* = 4), severe pancreatitis (*n* = 1), and a sudden unwitnessed event (*n* = 1)	18 (1–52.8)
Greenwood, 2021 [20]	NA	NA	HR: 0.85 (95% CI 0.36–2.10) for OS (*p* = 0.751)		44 (1–96)
Hough, 2016 [23]	NA	5-year EFS 16–24 years: 72.3% (66.2–78.4) 10–15 years: 83.6% (80.5–86.7) <10 years: 89.8% (88.4–91.2) OR = 2.1 (95% CI: 1.7–2.4), *p* (trend) < 0.00005, *p* (10–15 vs. ≥16) = 0.00004	5-year OS 16–24 years: 76.4% (70.5–82.3) 10–15 years: 87.5% (84.8–90.2) <10 years: 94.2% (93.2–95.2) OR = 2.7 (2.2–3.4), *p* (trend) < 0.00005, *p* (10–15 vs. ≥16) = 0.0004	5-year risk of DIR 16–24 years: 6.1% (2.8–9.4) 10–15 years: 3.4% (1.8–5.0) <10 years: 2.1% (1.5–2.7) OR = 2.0 (1.4–3.9), *p* (trend) = 0.0007 SAE incidence < 10 years vs. 10–24 years 2.58 (95% CI: 2.24–2.95), *p* < 0.00005 The time to first SAE was significantly shorter, and the cumulative incidence of SAEs was significantly higher in >10 years.	70 (1–121)
Valtis, 2022 [34]	NA	NA	NA	ON 5-year cumulative incidence (95% CI) < 30 years vs. 30–50 years: 21% (95% CI, 16–27) vs. 8% (CI, 4–14); univariate HR 2.77 (95% CI, 1.35–5.65); *p* = 0.004 ON 5-year cumulative incidence (95% CI) with peg-asparaginase vs. *E. coli* asparaginase 24% (95% CI, 18–30) vs. 5% (95% CI, 2–10); HR 5.28 (95% CI, 2.24–12.48); *p* = 0.001 ON 5-year cumulative incidence (95% CI): 15–19 years: 18 (12–25) 20–29 years: 25 (17–36) 30–39 years: 12 (5–23) 40–50 years: 4 (1–11) *p* = 0.003 (Gray test)	59 (1–169)
Toft, 2018 [32]	NA	5-year EFS (HR, 95% CI) 1–9 years: 0.89 ± 0.01 (ref.) 10–17 years: 0.80 ± 0.03 (2.0; 1.4–2.8) *p* < 0.001 18–45 years: 0.74 ± 0.04 (2.8; 2.0–4.0) *p* < 0.001	5-years OS (HR, 95% CI) 1–9 years: 0.94 ± 0.01 (ref.) 10–17 years: 0.87 ± 0.02 (2.3; 1.5–3.5) *p* < 0.001 18–45 years: 0.78 ± 0.03 (3.8; 2.5–5.7) *p* < 0.001	IRM 0.01 in all groups; *p* = 0.87 Adverse events in 1–9 vs. 10–17 vs. 18–45 years: No sig. difference in ICU^14^ admission, peripheral paralysis, anaphylactic reaction to ASP, invasive fungal infection, pancreatitis, hyperlipidemia, seizures Thrombosis: 3.6% vs. 15.3% vs.17.5% (*p* < 0.001) ON: 2.3% vs. 13.4% vs.8.5% (*p* < 0.001)	55 (36–77) 1–9 years: 59 10–17 years: 55 18–45 years: 38
Toft, 2016 [33]	NA	NA	NA	Increasing incidence of at least one toxic event (*p* < 0.0001): 1–9.9 years: 44.5% 10–14 years: 57.6% 15–17 years: 62.3% 18–26 years: 64.0% 27–45 years: 64.2% Toxic events during induction: There was no significant difference in ICU admissions, septic shock, heart failure, anaphylactic reactions, pancreatitis, seizures, coma, VOD, PRES, abdominal surgery, ON, liver or kidney dysfunction, bleeding, or peripheral paralysis. Hyperglycemia was more common >9 years (overall *p* < 0.0001) and 18–28 years (OR = 11.3 (95% CI: (2.9;43.5); *p* = 0.0002). Thrombosis was more frequent in 15–17 years (OR 10.2 (2.6;39.1), *p* = 0.0004) and 18–28 years (OR 7.3 (1.5;31.7), *p* = 0.007). Toxic events after induction: There was no significant difference in heart failure, pancreatitis, hyperglycemia, abdominal catastrophe, CNS catastrophe/bleeding, anaphylactic reaction, VOD, liver or kidney dysfunction, hypertension, *Pneumocystis jiroveci* pneumonia, PRES, coma, seizures, peripheral paralysis, or ICU admission. Increasing incidence of ON, thrombosis, and fungal infections with age (*p* < 0.0001, *p* < 0.0001, *p* = 0.006, respectively). OR (95% CI) for thrombosis was 5.4 (2.6–11.0), 5.1 (2.4–10.4), and 5.0 (2.2–10.8) for patients 15–17, 18–26, and 27–45 years, respectively, compared with children 1–9 years (all *p* < 0.0001). OR (95% CI) for avascular osteonecrosis for patients 10–14, 15–17, 18–26, and 27–45 years 10.4 (4.4–24.9, *p* < 0.0001), 6.3 (1.9–18.3, *p* = 0.001), 4.9 (1.3–15.0; *p* = 0.009), and 6.6 (1.8–21.2, *p* = 0.003) compared to 1–9 years, respectively.	40 (12–71)
Tantiworawit, 2019 [30]	NA		3-year OS (≤21 years vs. >21 years): 85% vs. 60%, *p* = 0.055	Toxicity (≤21 years vs. >21 years): There was no significant difference in the incidence of allergic reactions to ASP, pancreatitis, elevated liver enzymes or bilirubin, ON, thrombosis, stroke-like events, or neuropathy. Grade 3 hypofibrinogenemia: 10 vs. 21%, *p* = 0.006	40 (4–75)
Rytting, 2016 [29]	NA	NA	5-year OS ≤ 21 years (PIR vs. adult): 65% vs. 68%	NA	PIR: 66 months (17–107) Adult: 88 (1–152)
			5-year OS > 21 years (PIR vs. adult): 57% and 58% Differences between protocols were not statistically significant, and the *p*-value was not shown.		
Ribera, 2008 [28]	NA	EFS 15–18 vs. 19–30 years : 60% (95% CI : 43% –77%) vs. 63% (48%–78%), *p* = 0.97	OS 15–18 vs. 19–30 years: 77% (95% CI, 63%–91%) vs. 63% (46%–80%) *p* = 0.44	Toxicity (15–18 vs. 19–30 years): Grade 1 infections: 2.9 vs. 28%, *p* = 0.007 Grade 4 neutropenia: 44% vs. 59% Grade 4 thrombocytopenia: 10% vs. 33% Delays during reinduction were significantly more frequent in young adults than in adolescents, *p* = 0.04 Modifications in L-ASP or VCR were performed in 19% of cycles in adolescents vs. 33% in young adults, *p* = 0.03	50 (24–120)
Ribera, 2020 [27]	NA	5-year EFS 15–18 vs. 19–30 years: 78% (95% CI: 59–89) vs. 49% (31–65%), *p* = 0.151	5 years OS 15–18 vs. 19–30 years: 87% (95% CI: 74%–100%) vs. 63% (46%–80%), *p* = 0.021	There were no differences between adolescents and YA in drug modifications and delays	50 (0.5–114)
Quist-Paulsen, 2020 [25]	NA	5-year EFS (increasing age groups): 0.80 (95% CI: 0.72–0.88, ref.) vs. 0.75 (0.65–0.85) vs. 0.64 (0.52–0.76), *p*-values not shown	5-year OS (increasing age groups): 0.82 (95% CI: 0.74–0.88, ref.) vs. 0.76 (0.66–0.86, *p* = 0.3) vs. 0.65 (0.55–0.75, *p* = 0.01)	NA	Overall: 71 1–9 years: 76 (48–100) 10–17 years: 71 (53–91) 18–45 years: 68 (56–82)
Rank, 2018 [26]	NA	NA	NA	2.5-year cumulative incidence of any TE 1–9.9 years: 3.7% (2.64–4.8) 10–17.9 years: 15.5% (11.3–19.4) 18–45 years: 18.1% (13.2–22.8) *p* < 0.0001 he adjusted TE-specific hazard significantly increased in patients aged 6.0 to 14.9 years (HRa, 2.0; 95% CI, 1.2–3.5; *p* = 0.01), 15.0 to 20.9 years (HRa, 7.74; 95% CI, 4.52–13.2; *p* < 0.0001), and 21.0 to 45.9 years (HRa, 6.54; 95% CI, 3.69–11.6; *p* < 0.0001), using 1.0 to 5.9 years as reference. Patients aged 18.0–45.9: increased hazard of PE compared with children younger than 10.0 years (HRa, 11.6, 95% CI: 4.02–33.7; *p* < 0.0001). Adolescents aged 10.0 to 17.9 years: increased hazard of CSVT compared with children younger than 10.0 years (HRa 3.3, 95% CI: 1.5–7.3; *p* = 0.003).	52

CR: Complete remission; EFS: Event-free survival; RFS: Relapse-free survival; OS: Overall survival; IRM: Induction-related mortality; SAE: Severe adverse event; CALGB: Cancer and Leukemia Group B Study Group; DFCI: Dana–Faber Cancer Institute; DIR: Deaths in remission; ON: Osteonecrosis; ICU: Intensive care unit; PRES: Posterior reversible encephalopathy syndrome; VOD: Veno-occlusive disease/sinusoid obstruction syndrome; TE: Thromboembolism; PE: Pulmonary embolism; CSVT: Cerebral venous thrombosis; COG: Children’s Oncology Group; L-ASP: L-Asparaginase; VCR: Vincristine; AST: Aspartate aminotransferase; ALT: Alanine transaminase; NA: Not applicable; CI: Confidence interval; HR: Hazard ratio; AYA: Adolescents and young adults.

## Supplementary Materials

The supporting information about our search strategy and extracted data from each article can be downloaded at: https://www.mdpi.com/article/10.3390/curroncol30090625/s1. Supplementary S1: Search strategy; Supplementary S2: Extracted data of each included study.
