# Supplementary material for: Pediatric-Inspired Regimens in the Treatment of Acute Lymphoblastic Leukemia in Adolescents and Young Adults: A Systematic Review
Source: Curr Oncol. 2023 Sep 20;30(9):8612–32. doi: 10.3390/curroncol30090625 (PMC10528122; doi:10.3390/curroncol30090625)
Supplement: Supplementary file 1 [file curroncol-30-00625-s001.zip › curroncol-2529444-supplementary.pdf]

Pediatric-Inspired Regimens in the Treatment of Acute Lymphoblastic Leukemia in Adolescents and Young Adults: A Systematic Review

Aida Zeckanovic [1,2,*], Philipp Fuchs [1,2], Philip Heesen [3], Nicole Bodmer [1,2], Maria Otth [1,2,4,5,†] and Katrin Scheinemann [4,5,6,†]

Supplementary material

Supplementary S1: Search strategy

1. Cancer diagnoses in CAYA cancer patients	((leukemi*[tiab] OR leukaemi*[tiab]) AND (acute[tiab])) OR "Leukemia, Lymphoid"[Mesh]
2. "myeloid"	myeloid
3. Combine	#1 NOT #2
4. Different age categories	adolescen*[tiab] OR teenag*[tiab] OR (young adult*[tiab]) OR (young peopl*[tiab]) OR "Young Adult"[Mesh] OR Adolescent[Mesh]
5. Combine	#3 AND #4
6. Treatment	treatment*[tiab] OR "pediatric-like" OR "paediatric-like" OR "Antineoplastic Protocols"[Mesh]
7. Combine	#5 AND #6
8. Humans only	animals[Mesh] NOT humans[Mesh]
9. Combine	#7 NOT #8
10. Date	"2000/01/01"[Date - Publication] : "2022/10/01"[Date - Publication]
11. Combine	#9 AND #10

Supplementary S2: Extracted data for each included study

Advani A, et al. Comparison of CALGB 10403 (Alliance) and COG AALL0232 toxicity results in young adults with acute lymphoblastic leukemia
Blood advances, 2021

**Study design**<br>**Treatment era**	**Participants**	**Treatment**	**Main outcomes**	**Additional remarks**
☐ **Clinical trial**<br>**Observational**<br>☒ **Cohort**<br>☐ **Cross-sectional**<br>☐ **Other:**<br>__________<br><br>☐ **Retrospective**<br>☒ **Prospective**	Study population (N)<br>➢ Eligible cohort (registered):<br>CALGB 10403: 318<br>COG AALL0232: 926 (age 1-30 years)<br>➢ Analyzed cohort:<br>CALGB 10403: 289<br>COG AALL0232: 158 (aged 16-30 years)		☐ **Overall survival**<br>☐ **Event free survival**<br>☒ **Toxicity**	☒ **Age-stratified analysis**<br>☐ **Comparison to other age-groups mentioned**
<u>Centres:</u><br>Three cooperative groups: Alliance [Alliance for Clinical Trials in Oncology], SWOG [Southwest Oncology Group], and Eastern Cooperative Oncology Group [ECOG]<br><br><u>Country:</u><br>US<br><br><u>Treatment era:</u><br>CALGB 10403: 2007 – 2012<br>COG AALL0232: 2004 – 2011 (comparison)	<u>Inclusion criteria and cancer diagnosis:</u><br>B- or T-precursor ALL treated on CALGB 10403<br><br><u>Age at diagnosis:</u><br>Median (range) yr<br>CALGB 10403: 24 (18-39) yr<br>- 16-21 years: 33%<br>- 22-30 years : 45%<br>- 31-39 years : 22%<br>COG AALL0232: 17 (16-21) yr<br>- 16-21 years : 92%<br>- 22-30 years : 8%	<u>Name of protocol</u><br>CALGB 10403 (Alliance), NCT00558519= pediatric regimen<br>versus<br>COG AALL0232, NCT00075725 = pediatric regimen (using the arm identical to CALGB 10403; PC-arm) as comparison	<u>Definition of outcomes</u><br>- Grade 3 – 5 nonhematologic toxicity/events<br>- CTCAE version 3.0 for CALGB 10403<br>- CTCAE version 3.0 and later 4.0 for COG AALL0232<br><br><u>Main results (for analysis)</u><br>During induction<br>- Main Grade 3 and 4 toxicities with Incidence >15%: hyperglycaemia, ALT and bilirubin increase, febrile neutropenia, infection<br>- Mortality (p=0.34)<br>- CALGB 10403 3.1%: acute kidney injury (n=1), infection (n=1)<br>- COG AALL0232 1.3%: hepatic failure (n=2), sepsis (n=2), ventricular tachycardia (n=1), unknown (n=1), blood and lymphatic disorders (n=1), multiorgan failure (n=1), and nervous system disorders (n=1).<br><br>Post-remission toxicities and mortality<br>- Main Grade 3 and 4 toxicities with Incidence >15%: febrile neutropenia, infection, sensory neuropathy, hyperglycaemia, bilirubin, AST and ALT increase, anaphylaxis<br>- mucositis grade 3 or 4: CALGB 10403 9.1%, COG AALL0232 16.4% (p=0.037)	<u>Analysis</u><br>- descriptive statistics<br>- Chi-squared or Fisher's exact test<br>- p value ≤ 0.05 considered significant<br><br><u>Limitations:</u><br>- CALGB 10403: 61% completed intensive chemo; 39% completed all planned protocol treatment<br>- COG AALL0232: 57% of the AYA patients completed all therapy (74% of the patients <18 years of age)<br>No correlation between increased rate of serious (grade 3-4) toxicities and not completing treatment (toxicities not treatment limiting).<br><br><u>Strength:</u><br>Prospective study<br><br><u>Other considerations:</u><br>- hypersensitivity reaction with Asp decreased after CALGB 10403 protocol amendment to require premedication for

			- Mortality (p=0.64)<br>- CALGB 10403 1.3%<br>- COG AALL0232 0.8%<br><br>- trend (p=0.051) toward more delays in treatment (time from starting induction to beginning of maintenance) in CALGB 10403 (median, 64 days) compared with COG AALL0232 (59 days)<br><br><u>Age-stratified analyses</u><br>- 16-21 years, 22-30 years, and >31 years of age (Figure 1 and Figure 2)<br><br>Induction toxicities did not increase in frequency or severity with increasing age cohorts<br><br>Increased age correlated with a decreased fibrinogen level during induction and postremission therapy (odds ratios [ORs] of 1.103 [P =0.0001] and 1.111 [P =0 .0002], respectively) and elevated ALT during induction and postremission therapy (ORs of 1.037 [P = .039] and 1.045 [P=0 .011]).<br>But BMI might be a confounding factor for ALT.	PEG-Asp with corticosteroids, acetaminophen, and Diphenhydramine<br><br>Statistically significant difference in BMI by age group in COG AALL0232 (P 5 .037) but not in CALGB 10403. In both COG AALL0232 and CALGB 10403, patients with a lower BMI (<30 kg/m2) had a lower frequency of grade 3 to 5 toxicities (P 5 .002 for COG AALL0232).-→ BMI increases with age, so this is a potential confounding factor; no multivariate analysis shown.<br><br>*BMI as continuous variable was associated with an increased incidence of pancreatitis (OR, 1.078; P 5 .048), increased AST (OR, 1.072; P 5 .001), increased ALT (OR, 1.052; P 5 .001), and increased bilirubin (OR, 1.109; P , .0001) during induction. In addition, increased BMI was associated with an increased rate of AST (OR, 1.046; P 5 .006) and bilirubin (OR, 1.044; P 5 .025) during postremission therapy*<br><br>Quality (JBI: Cohort study): Quality 1

M. Al-Khabori, et al. Improved survival using an intensive, pediatric-based chemotherapy regimen in adults with T-cell acute lymphoblastic leukemia
Leukemia & Lymphoma, 2010

Study design<br>Treatment era	Participants	Treatment	Main outcomes	Additional remarks
☐ **Clinical trial**<br>**Observational**<br>☒ **Cohort**<br>☐ **Cross-sectional**<br>☐ **Other:**<br>__________<br><br>☒ **Retrospective**<br>☐ **Prospective**	Study population (N)<br>➢ Eligible/ Analysed cohort: 70<br>- 40 pat. with adult protocol<br>- 32 pat. with pediatric inspired protocol	**Adult protocols vs. Pediatric inspired DFCI**	☒ **Overall survival**<br>☐ **Event free survival → relapse free survival**<br>☐ **Toxicity**	☐ **Age-stratified analysis**<br>☐ **Comparison to other age-groups mentioned**
<u>Centres:</u><br>Princess Margaret Hospital/<br>University Health Network (PMH/UHN)<br><br><u>Country:</u><br>Canada<br><br><u>Treatment era:</u><br>January 1990 -March 2007	<u>Inclusion criteria and cancer diagnosis:</u><br>All T-ALL Patients aged <70 years who received induction therapy In PMH/UHN between 01/1990 - 03/2007<br>Five additional patients included with T-LBL (<25% blasts in bone marrow)<br><u>Age at diagnosis:</u><br>Median (range) 30.8 years (17–69 years)<br><br><u>follow-up:</u><br>Median (range)<br>54 months<br>(13–238 months)	<u>Name of protocol</u><br>Adult protocols (01/1990-06/2000)<br>- 9203ALL protocol (11 patients),<br>- Protocol C (7 patients),<br>- HyperCVAD (15 patients),<br>- MRC UKALL XII/ECOG E2993 protocol (7 patients)<br>Pediatric-inspired:<br>- DFCI regimen (32 patients)<br><br>Prior to December 2002, all patients in CR- 1 were offered alloSCT if an HLA-matched sibling was identified.<br>After December 2022 alloSCT was only offered in CR-2 or higher or if HR-features.	<u>Definition of outcomes</u><br>- OS: time from diagnosis to death or last follow up<br>- RFS: time from best response to death, relapse or last follow up<br><br><u>Main results (for analysis)</u><br>- 84% CR with DFCI protocol, 93% with non-DFCI protocols; p 0.7<br>- 3-year RFS: 89% in DFCI vs 24% in non-DCFI (p=<0.0001)<br>- 3-year OS: 81% in DFCI vs. 44% in non-DFCI (p=0.0003)<br>- 5-year OS: 75% (85% CI: 55–88%) in DFCI vs 25% (95% CI:13–39%) in non-DFCI group (p=0.0003).<br>- multivariate analysis including age, WBC, cytogenetics, CSF positivity, alloSCT in CR-1 and treatment group as variables; only the treatment group (DFCI vs. non-DFCI) was a significant for RFS (p=0.0001), only the treatment group (p=0.0008) and the CSF status (p=0.02) were significant for OS.<br><br>Mortality (descriptive only)<br>38 deaths overall	<u>Analysis</u><br>- comparisons of categorical variables: Fisher exact test, Pearson chi-square test<br>- continuous variables: Wilcoxon test<br>- survival probabilities: Kaplan–Meier method and log-rank test, with 95% CI<br>- Multivariate analysis with Cox Proportional-Hazard model<br>- significant if p-value <0.05<br><br><u>Limitations:</u><br>- retrospective, not randomized, low number of participants<br>- high treatment-related mortality associated with alloSCT maybe adversely influenced the OS in the non-DFCI group, but benefit persisted even if these patients were censored<br>- non-DFCI were treated prior to 2000 with less advanced supportive care (but without significantly more toxic deaths, thus probably not a big factor)

			- disease in 22 patients,<br>- infection in 8 patients (non-DFCI, five; DFCI, three),<br>- alloSCT-related complications (excluding infection) in 6 patients (all non-DFCI),<br>- other malignancy in 1 patient (non-DFCI),<br>- gastrointestinal bleed in 1 patient (DFCI).	JBI tool: Quality 1

Alacacioglu**, et al. Is the BFM Regimen Feasible for the Treatment of Adult Acute Lymphoblastic Leukemia? A Retrospective Analysis of the Outcomes of BFM and Hyper-CVAD Chemotherapy in Two Centers**
Chemotherapy, 2014

**Study design**<br>**Treatment era**	**Participants**	**Treatment**	**Main outcomes**	**Additional remarks**
☐ **Clinical trial**<br>**Observational**<br>☒ **Cohort**<br>☐ **Cross-sectional**<br>☐ **Other:** __________<br><br>☒ **Retrospective**<br>☐ **Prospective**	Study population (N)<br>➢ Analyzed cohort: 50<br>BFM-like N=20<br>Hyper CVAD n=30	BFM-Like vs. hyper-CVAD<br><br>+imatinib if Ph positive (4 pat. in each group)	☒ **Overall survival**<br>☐ **Event free survival → RFS**<br>☐ **Toxicity**	☐ **Age-stratified analysis**<br>☐ **Comparison to other age-groups mentioned**
Centres:<br>Katip Celebi and Dokuz Eylul Universities<br><br>Country:<br>Turkey<br><br>Treatment era:<br>March 2006 - October 2012	Inclusion criteria and cancer diagnosis: Adults diagnosed with ALL during the study period; inclusion criteria not clearly stated.<br><br>Age at diagnosis:<br>Median (range)<br>Overall: 27.5 years (range 18–59 years)<br>BFM group: median 25 years<br>Hyper-CVAD: median 30.5 years<br><br>follow-up (if applicable):<br>Median (range)<br>37 months	Name of protocol<br>BFM-Like vs. hyper-CVAD	Definition of outcomes<br>- OS: time between diagnosis and death (due to any causes) or the end of follow-up<br>- Relapse free survival (RFS): time from first remission to relapse or the end of follow-up.<br>- Toxicty<br><br>Main results (for analysis)<br>- CR rate after induction, 95% with BFM protocoll vs 96%<br>- OS mean: 41.5 ± 6.4 months in the hyper-CVAD group vs. 55.1 ± 4.9 in the BFM group, p = 0.012<br>- 5-year survival: 34% in hyper-CVAD vs 59% in BFM<br>- RFS: 39.1 ± 6.8 months in hyper-CVAD vs 53.9 ± 5.4 months in BFM, p = 0.009<br><br>- no anaphylactic reactions to *Escherichia coli* L -asparaginase, no pancreatitis attacks or venous complications. Elevations in liver enzymes were mild. No complications caused a delay in either protocol.	Analysis<br>- Descriptive for numerical variables: median, mean and range<br>- Descriptive for categorical variables: counts and relative frequencies.<br>- OS and RFS were estimated using the Kaplan-Meier product limit method<br>- P-value was two-sided, sig. <0.05<br><br>Limitations:<br>- Small number of patients, not randomized, retrospective<br>- Ph positivity in 25% of the BFM group but in only 13.3% of the hyper-CVAD group. In 5% of the hyper-CVAD group, the cytogenetic examinations were inadequate<br><br>JBI tool: Quality 2

Almanza-Huante**, et al.** Comparison of Two Pediatric-Inspired Regimens to Hyper-CVAD in Hispanic Adolescents and Young Adults With Acute Lymphoblastic Leukemia
Clinical Lymphoma, Myeloma & Leukemia, 2021

**Study design**<br>**Treatment era**	**Participants**	**Treatment**	**Main outcomes**	**Additional remarks**
☐ **Clinical trial**<br>**Observational**<br>☒ **Cohort**<br>☐ **Cross-sectional**<br>☐ **Other:**<br>__________<br><br>☒ **Retrospective**<br>☐ **Prospective**	Study population (N)<br>➢ Analyzed cohort: N=256<br>73 patients treated with pediatric-inspired regimen (46 modified versions of the ALL-BFM 90 and 27 CALGB C10403)<br>173 patients treated with hyper-CVAD.	**Pediatric inspired vs. adult protocol**	☒ **Overall survival**<br>☐ **Event free survival**<br>☐ **Toxicity**	☒ **Age-stratified analysis**<br>☐ **Comparison to other age-groups mentioned**
Centres:<br>Instituto Nacional de Cancerología, INCan and Instituto Nacional de Ciencias Médicas y Nutrición “Salvador Zubirán,” INCM<br><br>Country:<br>Mexico<br><br>Treatment era:<br>March 2016 - June 2019 for pediatric-inspired regimen<br><br>February 2009 -June 2015 for hyper-CVAD regimen	Inclusion criteria and cancer diagnosis:<br>newly diagnosed BCR-ABL1 negative ALL patients aged between 18 and 45 years<br>Age at diagnosis:<br>Median (range)<br>Overall median 22 (14-43) yr<br>Pediatric inspired; median 24 years<br>Hyper-CVAD: median 20 years<br><br>follow-up (if applicable):<br>Hyper-CVAD: median 8.4 years<br>BFM: median 2.7 years<br>CALGB; median 1.8 years	Name of protocol Pediatric inspired protocols: modified ALL-BFM 90 or modified CALGB C10403<br><br>Hyper-CVAD	Definition of outcomes<br>- CR < 5% blasts in bone marrow and hematologic recovery, defined as > 1000 neutrophils, > 100,000 platelets, and no transfusion requirements.<br>- Induction-related mortality (IRM): any death occurring after day 1 of induction therapy and before the next cycle<br>- Refractoriness: CR was not experienced after 2 cycles of induction<br>- OS: time in months from diagnosis until patient death or last follow-up<br>- Abnormal liver function test results: any value above 1.5 times the upper limit of normal<br><br>Main results (for analysis)<br>- 4-week CR rate: pediatric inspired 79.5% vs. hyper-CVAD 64.2% (p=0.02)<br>- 8-week CR rate: pediatric inspired 84.9% vs. hyper CVAD 73.7% (p = 0.06)<br>- IRM: pediatric inspired 1.4% vs. hyper-CVAD 8% (p=0.04)<br>- Relapse rate: pediatric inspired 44.1% vs hyper-CVAD 60% (p=0.04)<br>- Death: pediatric inspired 56.2% vs. 78.8% (p=<0.01)	Analysis<br>- chi-square test for categorical variables<br>- Kruskal-Wallis/Mann-Whitney U test for continuous variables.<br>- OS by Kaplan-Meier method and log-rank test for comparison<br>- Multivariate Cox proportional hazards regression: to evaluate independent prognostic factors associated with OS.<br>- sig. P <0.05<br>Limitations:<br>- Only 44.5% of the patients treated with hyper-CVAD completed the planned 8 courses.<br>- Median follow-up period of patients treated with the CALGB protocol was shorter than in the other protocols (BFM 32.4 vs. CALGB 21.8 months)<br>- patients treated with pediatric inspired protocols: marginally older than control group (median, 24 vs. 20 years; P < .01), lower CD20 expression (38% vs. 48.9%; P < .01), and frequent high-risk

			- Median OS: pediatric inspired 18.5 months [95% CI, 13.61-23.43] vs. hyper-CVAD 11.08 months [95% CI, 7.33-14.83])<br>- 24-month OS: pediatric inspired 41.5% vs. hyper-CVAD 28.1% (P =0 .01),<br>- The IRM and relapse rate were lower on PIR (1.4% vs. 8.0%; P = .04 and 44.1% vs. 60.0%; P = .02 respectively)<br><br>- Main cause of death in pediatric inspired and hyper-CVAD was disease progression (60.7% and 80%), followed by infections during CR (3.3% and 28.1%)<br><br>- Benefit of pediatric inspired only present in CALGB patients aged > 20 years (multivariate analysis). Eventually related to the higher toxicity of hyper-CVAD to older patients and less experience with BFM<br><u>Age-stratified analyses</u><br>- Patients < 20 years old (n =92)<br>4-week CR rate: 71.0% in pediatric inspired and 69.6% in hyper-CVAD (P = 1.0)<br>IRM: 0% in pediatric inspired and 10.1% in hyper-CVAD (P =0 .18)<br>median OS: 27.4 months (95% CI 9.5-45.3) in pediatric inspired and 15.4 months (95% CI 8.5-22.3) in hyper-CVAD (p=0.30)<br>- Patients > 20 years old (n = 118)<br>CR rate: was 57.4% in hyper-CVAD and 84% in pediatric inspired (P = .02)<br>IRM: 6.9% in hyper-CVAD and 2% in pediatric inspired (P = .39)<br>median OS: 9.2 months (95% CI 6-12.5) in hyper-CVAD versus 16.9 months (95% CI 13.1-20.6) in pediatric inspired (P < .01)	karyotypes (29.8% vs. 12.5%; P = .03).<br>- CALBG group had fewer patients with high-risk cytogenetics compared to the others.<br>- CAVE bias: all patients with pediatric inspired regimen treated in one hospital the others with Hyper-CVAD in the other<br><br><u>Other considerations:</u><br>CALGB cohort, that happens a few years later, result in a better survival curve that could be explained by better supportive therapy.<br><br>Benefit of PIR is underscored by the initial toxicity-associated IRM in the hyper-CVAD group<br><br>JBI Tool: Quality 1

			- Multivariate analysis of prognostic factors for OS:<br><20 years: hyperleukocytosis (HR ¼ 2.96; 95% CI, 1.40-6.26; P < .01), baseline abnormal liver function test results (HR ¼ 2.11; 95% CI,<br>1.04-4.28; P ¼ .04), ASCT (HR ¼ 0.17; 95% CI, 0.06-0.54; P < .01), and tumor lysis syndrome (HR ¼ 3.35; 95% CI, 1.31-8.59; P ¼ .01).<br><br>>20 years: receipt of CALGB regimen (HR ¼ 0.44; 95% CI, 0.20-0.97; P ¼ .04), ASCT (HR ¼ 0.52; 95% CI, 0.27-0.97; P ¼ .04), and experiencing 4-week CR (HR ¼ 0.22; 95% CI, 0.13-0.37; P < .01).	

Brandwein, et al. Predictors of outcome in adults with BCR-ABL negative acutelymphoblastic leukemia treated with a pediatric-based regimen
Leukemia Research, 2014

Study design<br>Treatment era	Participants	Treatment	Main outcomes	Additional remarks
☐ **Clinical trial**<br>**Observational**<br>☒ **Cohort**<br>☐ **Cross-sectional**<br>☐ **Other:**<br>__________<br><br>☒ **Retrospective**<br>☐ **Prospective**	Study population (N)<br>➢ Analyzed cohort: 156<br><br>17-<34 years: 73 (47%)<br>34-50 years: 54 (35%)<br>50-60 years: 29 (19%)	**DFCI 91-01 = pediatric like**<br><br><br>No comparison with adult protocol, but age-stratified analysis	☒ **Overall survival**<br>☐ **Event free survival**<br>☐ **Toxicity**	☒ **Age-stratified analysis**<br>☐ **Comparison to other age-groups mentioned**
Centres:<br>Princess Margaret Cancer Centre, University of Toronto<br><br>Country:<br>Canada<br><br>Treatment era:<br>June 2000 to June2011	Inclusion criteria and cancer diagnosis:<br>adults age 17–60 with BCR-ABL negative acute lymphoblastic leukemia treated with a pediatric-inspired protocol<br><br>Age at diagnosis:<br>Median (range) yr<br>37 (17–60)<br><br>follow-up (if applicable):<br>Median (range) yr<br>42 months (range 0.3–135 months)<br><br>For surviving patients<br>56 months (range 13–135 months)	Name of protocol<br>modified Dana Farber Consortium (DFCI) 91-01 protocol<br><br>HSCT (allo/auto)<br>Prior to December 2001, patients with an HLA matched sibling donor in CR1 were referred for allogeneic HSCT. Later HSCT in CR1 just for high-risk patients.	Definition of outcomes<br>- CR: <5% blasts in a normocellular marrow, with ANC > 1.0 × 109/L, platelets > 100 × 109/L and no evidence of extramedullary disease.<br>- OS: time from initial diagnosis until death or last follow-up<br>- Disease-free survival (DFS): time from achievement of CR until, relapse, death or last follow-up<br><br>Main results (for analysis)<br>- CR 93% (145/156)<br>- 5-year OS: 66% (95% C.I. 57–73%),<br>- 5-year DFS: 70% (95% C.I. 61–77%)<br><br>- 18 patients died in CR1 due to other causes – these included HSCT-related complications (7 patients), sepsis on chemotherapy (5), intracranial hemorrhage (1), CNS masses of unknown etiology (1), metastatic breast cancer (1), secondary AML (1) and unknown causes	Analysis<br>- Descriptive: counts, percentage, mean, median, range, SD<br>- Kaplan–Meier method for OS, DFS and cumulative incidence o relapse; Log-rank test to compare survival distributions<br>- Cox proportional hazards regression to assess the effect of potential predictors in univariate analysis<br>- p-values 2-sided; p < 0.05 was significant<br>Limitations:<br>- retrospective, low number of patients in each age group<br><br>Other considerations:<br>There was a trend toward a higher cumulative incidence of relapse (CIR) in patients who received <80% of the planned asparaginase dose during intensification; however this difference was not statistically significant (5 year CIR 20.5% vs. 32.7%,p = 0.09).

			Age-stratified analyses<br>- 5-year OS (95%CI), univariate analysis:<br>- 17-34 years: 80% (67-88%)<br>- 34-50 years: 50% (35-63%)<br>- 50-60 years: 62% (42-77%)<br>P=0.001<br>- CR<br>- 17-34 years: 99%<br>- 34-50 years: 87%<br>- 50-60 years: 90%<br>P=0.02<br>- age (cont. variable) was an independent significant predictor of OS: p=0.0046 in all patients and B-ALL only p=0.0029<br>- 5-year OS age < 34 and low WBC (n = 57): 85% (95%C.I. 71–93%); age <34 and high WBC (n = 15): 57% (28–78%); age > 34 and low WBC (n = 73): 57% (44–68%); age > 34 and high WBC (n = 10): 30% (7–58%), p=0.0001	JBI Tool: Quality 1

Burke, et al. Outcomes in adolescent and young adult patients (16 to 30 years) compared to younger patients treated for high-risk B-lymphoblastic leukemia: Report from Children's Oncology Group Study AALL0232
Leukemia, 2022

**Study design**<br>**Treatment era**	**Participants**	**Treatment**	**Main outcomes**	**Additional remarks**
☐ **Clinical trial**<br>**Observational**<br>☒ **Cohort**<br>☐ **Cross-sectional**<br>☐ **Other:**<br>__________<br><br>☐ **Retrospective**<br>☒ **Prospective**	Study population (N)<br>➢ Original cohort: 3,154<br>➢ Eligible cohort: 3,040<br>➢ Analyzed cohort:<br><16 yeras: n=2,443 (=younger population)<br>16-30 years: n= 597 (=AYA population)	**COG** AALL0232<br><br>No comparison with adult protocol, but age-stratified analysis	☒ **Overall survival**<br>☒ **Event free survival**<br>☐ **Toxicity**	☒ **Age-stratified analysis**<br>☒ **Comparison to other age-groups mentioned**
Centres:<br>COG Centers<br><br>Country:<br>USA<br><br>Treatment era:<br>January 2004 - January 2011	Inclusion criteria and cancer diagnosis:<br>newly diagnosed HR B-ALL (1–9 years old with an initial WBC ≥50,000/microliter or 10–30 years old with any WBC), patients with DS excluded<br><br>Age at diagnosis:<br>Median (range) yr of AYA population<br>17 years (16-30)<br><br>Follow-up (if applicable):<br>Median (range) yr<br>NA	Name of protocol<br>**COG** AALL0232	Definition of outcomes<br>- EFS: time from study entry to first event (Induction failure, Induction death, relapse, second malignancy, remission death), or date of last follow-up<br>- OS: time from study entry to death or date of last follow-up<br>-Toxicty reported according to CTCAE version 3.0 and version 4.0 (after Dec. 2010)<br>-<br><br>Main results (for analysis)<br>- 5-year EFS rate 65.4±2.2% for AYA vs. 78.1±0.9% for younger patients (p<0.0001)<br>- 5-year OS rates 77.4±2.0% for AYA vs. 87.3±0.7% for younger patients (<0.0001)<br>- After excluding VHR B-ALL: 5-year EFS (67.3±2.3% for AYA vs. 80.5±0.9% for younger patients, p<0.0001) and OS (79.7±2.0% for AYA vs. 89.3±0.7% for younger patients, p<0.0001)<br>- Induction death: 2.2% in AYA versus 1.6% in younger (p=0.366)	Analysis<br>- Comparison <16 years and >16 years based on receiver-operating characteristic (ROC) analysis incorporating EFS and age (limited to those ≥10 years) ; HR=1.51, [1.274, 1.784]; p=0.0000018<br>- Kaplan-Meier method for survival curves, comparison with log-rank test.<br>- Multivariable Cox regression analyses of outcomes<br>- Cumulative incidence rates for relapse, secondary malignant neoplasm (SMN), and remission deaths.<br>- Comparisons with K-sample test for comparison of cumulative incidences<br>- p-value < 0.05 was considered as significant<br>Strengths<br>-a prospective study with many analyzed patients<br>Limitations:

			- 5-year cumulative incidence rate of relapse; 18.5±1.7% in AYA versus 13.5±0.7% in younger patients, p=0.0006<br>- therapy completion rate: 50.3% in AYA vs 65.8% in younger (p<0.0001)<br>o 22.6% of AYA and 15.5% of younger patients due to on-therapy event<br>o 6.4% of AYA and 4.9% of younger patients due to VHR ALL features at the end of induction<br>o 20.7% of AYA and 13.8% of younger patients toxicity, patient/family refusal, and physician's choice.<br>Univariate Cox Regression Analysis for EFS (HR, 95%CI)<br>- Age <16 vs >16 years: 0.558 (0.469, 0.663), p<0.0001<br>- Age continuous: 1.060 (1.045, 1.075), p<0.0001<br><br>Multivariable Cox Regression Analysis for EFS<br>- Age <16 vs >16 years: 0.773 (0.625, 0.956), p=0.018<br>- Age continuous: 1.042 (1.024, 1.060), p<0.0001<br><br>Toxicity Grade ≥3 in induction (AYA vs. younger)<br>- hyperglycemia: 23.6% vs. 15.4% (p<0.0001)<br>- hyperbilirubinemia: 6.9% vs 3.7% (p=0.0007)<br>- febrile neutropenia: 7.4% vs, 13.8% (p=<0.0001)<br>- thrombosis: 1.5% in AYA vs. 1.2% in younger (p=0.470)<br>- pancreatitis: 0.5% in AYA vs. 0.5% (p=0.972)<br>Toxicity Grade ≥3 in post-induction (AYA vs younger))<br>- mucositis: 18.2% vc. 11.7% (p=0.0002)	- unclear what was used in multivariable analysis<br><br>Other considerations:<br>AYAs more likely to have the Ph-like ALL gene expression profile (17.7% versus 11.5%, p=0.015) and less likely to have ETV6-RUNX1 fusion (3.8% versus 16.4%, p<0.0001).<br><br>Sub-analysis on obesity: 4.8% obese subject in 1–15 age group vs. 19.5% in AYA (p<0.001). Rates of many toxicities higher in obese versus non-obese patients. However, rates of toxicities similar between obese AYA and younger patients. Obese AYA patients had significantly lower 5-year EFS (50.8±5.4% (n=115) as compared to obese younger patients 66.9±4.8% (n=117); p=0.006)<br><br>JBI Tool: Quality 1

			- peripheral neuropathy: 12.1% vs.7.8% (p=0.001)<br>- <u>febrile neutropenia: 45.2% vs 56.8% (p=<0.0001)</u><br>- hyperbilirubinemia: 17.3% vs. 9.5% (p<0.0001)<br>- hepatic failure: 1.3% vs. 0.3% (p=0.009)<br>- Deaths in remission 5.7% vs. 2.4%, (p<0.0001), mostly Grade 5 infections<br><br><u>Age-stratified analyses</u><br>Analysis ≥22 years vs. 16 to 21 years showed no difference in rates of relapse (15.0±5.7% versus 18.8±1.8%, p=0.88) or deaths in remission (5.5±1.0% versus 7.5±4.2%, p=0.69)	

Chao-Neng Cheng**, et al.** Outcome of young adult patients with very-high-risk acute lymphoblastic leukemia treated with pediatric-type chemotherapy e a single institute experience<br>Journal of the Formosan Medical Association, 2022				
**Study design<br>Treatment era**	**Participants**	**Treatment**	**Main outcomes**	**Additional remarks**
☐ **Clinical trial**<br>**Observational**<br>☒ **Cohort**<br>☐ **Cross-sectional**<br>☐ **Other:**<br>__________<br><br>☒ **Retrospective**<br>☐ **Prospective**	Study population (N)<br>➢ Original cohort:<br>35 patients with ALL<br>➢ Eligible cohort:<br>27 patients with VHR ALL<br>- 11 Hyper-CVAD/HD-MTX and Ara-C<br>- 16 TPOG		☐ **Overall survival**<br>☒ **Event free survival**<br>☐ **Toxicity**	☒ **Age-stratified analysis**<br>☐ **Comparison to other age-groups mentioned**

<u>Centres:</u><br>National Cheng Kung University Hospital<br><br><u>Country:</u><br>Taiwan<br><br><u>Treatment era:</u><br>2008 -2019	<u>Inclusion criteria and cancer diagnosis:</u><br>VHR ALL patients aged between 18 and 40 years at diagnosis<br><br><u>Age at diagnosis:</u><br>Median (range)<br>TPOG: 24.3 years (18-36)<br>Hyper-CVAD: 33 years (20-40)<br><br><u>Age at follow-up (if applicable):</u><br>Median (range)<br>TPOG 60 months (6-108)<br>Hyper CVAD 20 months (2-127)	<u>Name of protocol</u><br>Taiwan Pediatric Oncology Group-Acute Lymphoblastic Leukemia-2002<br>(TPOG-ALL-2002) protocol versus Hyper- CVAD/HD-MTX and Ara-C<br><br>+ Dasatinib for Ph+ ALL	<u>Definition of outcomes</u><br>- EFS: time from diagnosis to an event (relapse or death) or last follow-up<br><u>Main results (for analysis)</u><br>- 5-year EFS: 71.6 +/- 12.2% in TPOG versus 45.5 +/- 15.0% in Hyper CVAD (p=0.152); Hazard ratio of 0.42 (p=0.16)<br>- Toxic death: n=1 in both groups<br>- Relapse: n=4 in TPOG versus n=5 in Hyper-CVAD<br>- 5-year EFS in untransplanted patients: 28.6%+/- 17.1% for hyper-CVAD vs. 83.3% +/- 10.8% for TPOG; HR 4.19, p < 0.05<br><u>Age stratified analysis for patients treated with pediatric-inspired protocol</u><br>- 5-year EFS (univariate): age <25 years 76.2% +/-14.8% versus age ≥25 years 64.3% +/-21.0% (p=0.265)	<u>Analysis</u><br>- Differences in categorical variables: Fisher's exact or ChieSquare tests<br>- Differences in continuous variables: ManneWhitney test<br>- KaplaneMeier method or EFS and comparison by log-rank test and Cox proportional hazard model<br><u>Limitations:</u><br>Very small number of patients<br><br><u>Strength:</u> children on pediatric protocol treated by pediatric oncologist<br><br><u>Other considerations:</u><br>- importance of MRD-guided strategy for HSCT indications<br><br>JBI Tool: Quality 1

**DJ DeAngelo, et al.** Long-term outcome of a pediatric-inspired regimen used for adults aged 18–50 years with newly diagnosed acute lymphoblastic leukemia<br>Leukemia, 2015				
**Study design**<br>**Treatment era**	**Participants**	**Treatment**	**Main outcomes**	**Additional remarks**
☒ **Clinical trial**<br>**Observational**<br>☒ **Cohort**<br>☐ **Cross-sectional**<br>☐ **Other:**<br>__________<br><br>☐ **Retrospective**	Study population (N)<br>➢ Original cohor: 100<br>➢ Eligible cohort: 92<br>➢ Analyzed cohort: 92 (57 for L-Asp)<br>18-29 years: 48 (52%)<br>30-50 years: 44 (48%)		☒ **Overall survival**<br>☐ **Event free survival**<br>☐ **Toxicity**	☒ **Age-stratified analysis**<br>☐ **Comparison to other age-groups mentioned**

☒ **Prospective**				
Centres:<br>13 participating centers<br><br>Country:<br>USA, Canada<br><br>Treatment era:<br>August 2002- February 2008	Inclusion criteria and cancer diagnosis:<br>patients aged 18–50 years with ALL (excl. mature B-cell ALL) and a Zubrod performance status of 2 or less<br><br>Age at diagnosis:<br>Median (range) yr<br>28 years (18-50)<br><br>follow-up (if applicable):<br>Median (range) yr<br>4.5 years (95% CI 4.1–5.0 years)	Name of protocol<br>DFCI Pediatric ALL Consortium regimen/ DFCI Adult ALL Consortium Protocol 01–175<br><br>+ Imatinib for Ph+ ALL from Sept. 2006	Definition of outcomes<br>- primary end point: proportion of patients who completed 30 weeks of asparaginase treatment (feasibility study)<br>- Outcome events: death during induction therapy, failure to achieve CR at the end of 4-week induction phase, death during remission and relapse.<br>- EFS: time from study registration to the first outcome event.<br>- Disease-free survival (DFS): time from CR to relapse or death (only patients who achieved a CR included)<br>- OS: time from study registration to the time of death from any cause<br>- Patients not experiencing an outcome event were censored at date of last follow-up<br>- Patients after HSCT not censored at the date of transplant,<br><br>Main results whole cohort<br>- 4-year OS 67% (95% CI 56–76%),<br>- 4-year DFS 69% (95% CI 56–78%)<br>- 78 (85%) (90% exact CI: 77–91%) achieved a CR at the end of the 4 weeks<br><br>age-stratified analyses<br>- 4-year DFS % (95%CI): age 18-20 years 70% (52-83%) versus age 30-50 years 67% (50-79%), p 0.54<br>- 4-year EFS % (95%CI): age 18-20 years 55% (39-69%) versus age 30-50 years 61% (44-74%), p 0.61	Analysis<br>- Kaplan–Meier method for EFS, DFS, and OS; and the Greenwood formula to construct 95% CI.<br>- Univariate analyses of differences in EFS, DFS and OS by log-rank tests.<br>- The Common Terminology Criteria for Adverse Events (CTCAE) version 2.0 to code toxicities.<br><br>Strength:<br>Prospective study<br><br>JBI Tool: Quality 2

			- 4-year OS % (95%CI): age 18-20 years 68% (52-80%) versus age 30-50 years 65% (49-77%), p 0.93	

Ganesan, et al. Outcomes in adolescent and young adult acute lymphoblastic leukaemia: a report from the Indian Acute Leukaemia Research Database (INwARD) of the Hematology Cancer Consortium (HCC)
British Journal of Haematology, 2021 (correspondence)

**Study design**<br>**Treatment era**	**Participants**	**Treatment**	**Main outcomes**	**Additional remarks**
☐ **Clinical trial**<br>**Observational**<br>☒ **Cohort**<br>☐ **Cross-sectional**<br>☐ **Other:**<br>☒ **Retrospective**<br>☐ **Prospective**	Study population (N)<br>➢ Original cohort:<br>1383 registered<br>➢ Eligible cohort:<br>1141 received treatment<br>➢ Analyzed cohort:<br>**Adult protocols (n=139)**<br>**Paediatric protocols (n=1002)**	Pediatric type:<br>- Multicentre protocol 841 (MCP-841),<br>- Berlin-Frankfurt- Münster 95 (BFM-90, -95 or -2000),<br>- Children's Oncology Group (COG)<br>Adult type<br>- German Multicentre ALL (GMALL),<br>- Hyper-CVAD,<br>- UKALL	☒ **Overall survival**<br>☒ **Event free survival**<br>☐ **Toxicity**	☒ **Age-stratified analysis**<br>☐ **Comparison to other age-groups mentioned**
Centres:<br>Data from retrospective database maintained by the Hematology Cancer Consortium (HCC).<br><br>Country:<br>India<br><br>Treatment era:<br>2012 – 2017	Inclusion criteria:<br>All ALL patients included in the database in the given time period who underwent treatment<br><br>Cancer diagnosis:<br>ALL (including B and T-ALL and MPAL)<br><br>Age at diagnosis:<br>Median (range) yr<br>Whole cohort: range 15–29 years<br>Paediatric type protocols median age 20; mean + SD 20.5 ± 4.1<br>Adult type 23 years, mean + SD 20.9 ± 4.2<br><br>follow-up (if applicable):	Name of protocol<br>BFM n = 846 (74%)<br>COG n= 97 (9.7%)<br>MCP-841 n=42 (4.2%)<br>Other pediatric n=6 (0.6%)<br><br>GMALL n= 108 (77.7%) + 11 cases classified as pediatric<br>Hyper-CVAD n=26 (18.7%)<br>Other adult: n=5 (3.6%)	Definition of outcomes<br>- EFS<br>- OS<br>- RFS<br><br>Main results (for analysis)<br>- Pediatric versus adult protocol (pediatric=ref.) as HR (95%CI)<br>- EFS: 1.05 (0.81-1.35), p=0.736)<br>- OS: 1.72 (1.29–2.29), p<0.001 (univariate)<br>- OS: 3.19 (1.95-5.22), p<0.001 (multivariate)<br>➔ worse in adult protocol<br>- 2-year EFS: pediatric 56.6% versus aldult 52.1%; p=0.730<br>- 2-year OS: pediatric 75.4% versus adult 59.0%; p<0.001<br>- 2-year RFS: pediatric 75.1% versus adult 75.4%; p=0.702	Analysis<br>-Kaplan–Meier estimates<br>- Cox Proportional Hazard Regression model for multivariate analysis<br>- stat. test used for univariate analysis not specified<br><br>Limitations:<br>- Retrospective, large majority of patients belong to the pediatric group<br>- not randomized<br>- Median age differs between pediatric type and adult type protocols (20 vs. 23 years, P = 0_001); mean age not significantly different between groups<br>Strength:<br>- multivariate analysis<br><br>Other considerations:

	Median 23 months [95% confidence interval (CI) 6–38]		<u>Age-stratified analyses</u><br>- EFS as HR (95%CI):<br>- 15-17 years=ref.<br>- 18-24 years=1.01 (0.83-1.23), p=0.937<br>- 25-29 years= 1.02 (0.80-1-30); p=0.862<br>- OS as HR (95%CI)<br>- 15-17 years=ref<br>- 18-24 years=1.20 (0.91-1.58), p=0.203<br>- 25-29 years=1.37 (0.99-1.89), p=0.057<br>- 2-year EFS<br>- 15-17 years: 56.7%<br>- 18-24 years: 55.9%<br>- 25-29 years: 55.4%<br>P=0.984<br>- 2-year OS<br>- 15-17 years: 76.6%<br>- 18-24 years: 73.0%<br>- 25-29 years: 69.3%<br>P=0.153<br>- 2-year RFS<br>- 15-17 years: 74.8%<br>- 18-24 years: 75.3%<br>- 25-29 years: 75.4%<br>P=0.948<br>Toxicity not specified by protocol.	- OS better post 2014 in univariate and multivariate analysis (post 2014 pediatric protocols more commonly used). Better outcomes might be because of better supportive treatment, leukemia characteristics or pediatric protocol.<br><br>JBI Tool: Quality 1

Ganesan P, et al. Acute Lymphoblastic Leukemia in Young Adults Treated with Intensive ''Pediatric'' Type Protocol
Indian Journal of Hematology and Blood Transfusion, 2018

**Study design**<br>**Treatment era**	**Participants**	**Treatment**	**Main outcomes**	**Additional remarks**
☐ **Clinical trial**<br>**Observational**<br>☒ **Cohort**<br>☐ **Cross-sectional**<br>☐ **Other:**<br>__________<br><br>☒ **Retrospective**<br>☐ **Prospective**	Study population (N)<br>➢ Original cohort:<br>➢ Analyzed cohort: 232 Patients		☒ **Overall survival**<br>☒ **Event free survival**<br>☒ **Toxicity**	☐ **Age-stratified analysis**<br>☐ **Comparison to other age-groups mentioned**
<u>Centres:</u><br>Cancer Institute (WIA),<br>Chennai, Tamilnadu<br><br><u>Country:</u><br>India<br><br><u>Treatment era:</u><br>January 2000 - December 2014	<u>Inclusion criteria and cancer diagnosis:</u><br>Young adults (18–30 years) with Ph-negative ALL treated<br>in the given time period.<br><br><u>Age at diagnosis:</u><br>Median (range)<br>21 years (18–30)<br><br>BFM vs. other mean 21.8 years vs. 22.4 years, p = 0.218<br><br><u>Follow-up (if</u><br>Median 21 months (range 0.3–165 months),<br><br>Patient subgroup who are still alive: median 48 months	<u>Name of protocol</u><br>BFM 95: N = 147 (63%),<br><br>Adult protocols:<br>- MCP-841: N = 51 (22%)<br>- GMALL: N = 21 (9%)<br>- INCTR: N = 9 (4%)<br>- UKALL: N = 4 (2%)<br><br>No patient received allogenic transplant in CR1.<br><br>**<u>No stratification based on baseline risk status or on MRD</u>**<br>**<u>Studies in BFM</u>** (all patients received the SR Arm with HD-MTX 5 g/m2 during consolidation).<br>The induction dose of L-asparaginase was reduced<br>from 10,000 units/m2 to a uniform dose of 10,000 units per patient from 2013	<u>Definition of outcomes</u><br>- CR: recovery of counts (neutrophils[1000/cmm and platelet counts[100,000/cmm) with\5% blasts in the marrow by morphology<br>- Relapse-free survival (RFS): from documentation of CR till relapse or death<br>- Event-free survival (EFS): from start of treatment till last follow-up or failure to achieve remission after induction, relapse, or death due to any cause.<br>- Overall survival (OS): from start of treatment till death due to any cause<br><br><u>Main results (for analysis)</u><br>- CR: 84% in BFM vs 82% in other protocols<br>- 5-yr RFS: 51% in BFM versus 35% in others (p = 0.027)<br>- 5-year OS: 43% in BFM versus 33% in others (p = 0.2)<br>- 5.year EFS: 40% in BFM versus 27% in others (p=0.054)<br>- Mortality during induction: 10% in BFM-95 versus 1% in other protocols (p	<u>Analysis</u><br>- Kaplan–Meier method and comparison by log-rank test<br><br><u>Limitations:</u><br>- not randomized<br>- no risk-adapted treatment for BFM protocol<br>- significant treatment delays in BFM (median duration 9 months; expected 6-7 months)- thus worse outcomes; reasons probably multifactorial, not investigated in the study<br>- outcomes poorer that those reported in literature for age groups; perhaps high mortality partially due to suboptimal supportive treatment and financial/insurance issues<br><br><u>Strength:</u><br><br><u>Potential bias/methodological problems:</u><br><br><u>Other considerations:</u>

			= 0.001); major causes of death were sepsis and L-asparaginase associated thrombotic complications<br>- Treatment-related deaths: 12% (18/147) in BFM protocol versus 2% (2/85) in other protocols (p = 0.031)	improvement came at the cost of increased incidence of treatment-associated deaths (12 vs. 2%) which negated any impact on the OS<br><br>JBI Tool: Quality 2

Gómez-De León**, et al.** Treatment of Ph-Negative Acute Lymphoblastic Leukemia in Adolescents and Young Adults with an Affordable Outpatient Pediatric Regimen<br>*Clinical Lymphoma, Myeloma and Leukemia, 2022*				
**Study design**<br>**Treatment era**	**Participants**	**Treatment**	**Main outcomes**	**Additional remarks**
☐ **Clinical trial**<br>**Observational**<br>☒ **Cohort**<br>☐ **Cross-sectional**<br>☐ **Other:** __________<br><br>☐ **Retrospective**<br>☒ **Prospective**	Study population (N)<br>➢ Original cohort: 105<br>➢ Eligible cohort: 91<br>➢ Analysed cohort: 66 at the end of induction; 39 remained in follow up		☒ **Overall survival**<br>☒ **Event free survival**<br>☐ **Toxicity**	☒ **Age-stratified analysis**<br>☐ **Comparison to other age-groups mentioned**
Centres:<br>Universidad Autónoma de Nuevo Léon, Hematology Service, Monterrey<br><br>Country:<br>Mexico<br><br>Treatment era:<br>2016 to 2020	Inclusion criteria and diagnosis:<br>BCR-ABL negative B-ALL, aged 16-45 years<br>Exclusively patients without access to private insurance who must cover for the cost of care out of pocket.<br><br>Age at diagnosis:<br>Median (range)<br>21 years (15-45)<br><br>Follow-up (if applicable):<br>Median (range)<br>18 months (1-52.8)- in surviving patients	Name of protocol<br>Modified pediatric Berlin-Frankfurt-Münster schema (more affordable and given in an out-patient setting)	Definition of outcomes<br>- CR: marrow aspirate with < 5% blasts and hematopoietic recovery with an absolute neutrophil and platelet count >1 ×10 9 /L and 10×10 9 /L, respectively<br>- EFS: period without refractory disease, relapse, or death due to any cause.<br>- OS: period between diagnosis and death due to any cause.<br>- Leukemia-free survival (LFS): time from CR with events being relapse or death in CR.<br>- Treatment abandonment (TA): arbitrarily defined as a missed ≥14-day period during intensive treatment or ≥1 month during maintenance<br><br>Main results (for analysis)<br>Whole cohort: OS at 24 months was 61.5%, EFS 49.8% and LFS 54.3%; MRD neg. with better outcomes<br><br>Age-stratified analyses<br>- Patients ≥40 years (n = 11) had worse EFS and LFS compared to younger	Analysis<br>- Descriptive<br>- Categorical variables: chi-square or Fisher's exact test<br>- Continuous variables: Student's t'test or the Mann-Whitney U<br>- Survival outcomes by Kaplan-Meier method and comparison by log-rank test as intent-to-treat<br><br>Limitations:<br>- small sample size, a high attrition rate, and a short follow-up<br>- 52/91 patients did not complete the intensive treatment phase<br>o relapsed or refractory disease (n = 13; 14.3%),<br>o treatment-related mortality or abandonment (n = 12; 13.2% in both cases),<br>o institution transfers (n = 11; 12.1%),<br>o change in regimen due to toxicity or residual

			patients with a median of only 8.3 months (95% CI 0-21.2; *P* = .006) and 7 months, respectively (95% CI 0-14.4; *P* = .03) without a statistically significant difference in OS<br>- Univariable analysis for age by HR(95%CI)<br>- OS: 1.03 (0.9-1.07)<br>- EFS: 1.93 (0.99-1.07)<br><br>Toxicity (descriptive only):<br>causes of death during induction were infectious complications (n = 4), CNS thrombosis or bleeding (n = 4), severe pancreatitis (n = 1), and a sudden unwitnessed event (n = 1)	disease (n = 2; 2.2% in both)<br>- treatment abandonment 26.4%<br><u>Sterngths:</u><br>- standardized approach, prospective, use of MRD<br><br>JBI Tool: Quality 2

Greenwood**, et al.** An MRD-stratified pediatric protocol is as deliverable in adolescents and young adults as in children with ALL
Blood advances, 2021

**Study design**<br>**Treatment era**	**Participants**	**Treatment**	**Main outcomes**	**Additional remarks**
☐ **Clinical trial**<br>**Observational**<br>☒ **Cohort**<br>☐ **Cross-sectional**<br>☐ **Other:**<br>__________<br><br>☐ **Retrospective**<br>☒ **Prospective**	Study population (N)<br>➢ Original cohort: 86<br>➢ Eligible cohort: 82		☒ **Overall survival**<br>☐ **Event free survival**<br>☐ **Toxicity**	☒ **Age-stratified analysis**<br>☐ **Comparison to other age-groups mentioned**
Centres:<br>15 centers in Australia<br><br>Country:<br>Australia<br><br>Treatment era:<br>July 2012 – June 2018	Inclusion criteria and cancer diagnosis:<br>patients aged 15 to 39 years with B- or T-cell ALL<br><br>Age at diagnosis:<br>Median (range)<br>22.7 years (16-38 years)<br><br>Follow-up (if applicable):<br>Median (range)<br>44 months (1-96 months)	Name of protocol<br>Australasian Leukaemia and Lymphoma Group (ALLG) ALL06 study: based on the ANZCHOG Study 8 protocol<br>and consisted of AIEOP-BFM 2000 treatment blocks	Definition of outcomes<br>- Primary end point: percentage of participants starting protocol M or HR1 by day 94<br>- Complete remission (CR): no morphologic evidence of leukemia cells in peripheral blood and <5% blasts in BM aspirate and no evidence of extramedullary disease<br>- Relapse: presence of identifiable leukemic cells in peripheral blood on blood film, >5% blasts in BM aspirate, or recurrence of extramedullary disease<br>- DFS: from CR until the date of relapse or death<br>- OS: from day 1 of protocol treatment to death<br><br>Main results (whole cohort)<br>- 3-yr DFS 72.8% (95% CI, 62.8-82.7)<br>- 3-yr OS 74.9% (95% CI, 65.3-<br>84.5).<br><br>Age-stratified analyses<br>- Age (less than the median) as univariate predictor of survival: HR 1.01 (95%CI	Analysis<br>- Kaplan-Meier for DFS and OS; 95% CIs calculated by using Greenwood's formula<br>- Cox proportional hazards regression models for univariate and multivariate to evaluate associations between demographic and clinical features and OS and DFS<br>- Student t tests or Mann-Whitney U test<br>- Poisson approximation for event rates and Fisher's exact tests for adverse events<br><br>Limitations: small nr. of patients<br><br>Strength: prospective<br><br>JBI Tool: Quality 1

			1.44-2.33) for DFS (p=0.985) and HR 0.85 (95%CI 0.36-2.10) for OS (p=0.751)	

**Gupta S, et al. The effect of adopting pediatric protocols in adolescents and young adults with acute lymphoblastic leukemia in pediatric vs adult centers: An IMPACT Cohort study**<br>Cancer science, 2019				
**Study design<br>Treatment era**	**Participants**	**Treatment**	**Main outcomes**	**Additional remarks**
☐ **Clinical trial**<br>**Observational**<br>☒ **Cohort**<br>☐ **Cross-sectional**<br>☐ **Other:**<br>__________<br><br>☒ **Retrospective**<br>☐ **Prospective**	Study population (N)<br>➢ Original cohort: 275<br>➢ Analyzed cohort:<br>152 treated in adult center<br>123 treaed in pediactic cent.<br>59 treated at adult centers with pediatric protocols<br>20 treated at adult centers with adult protocols<br>44 treated at pediatric centers with pediatric protocols		☒ **Overall survival**<br>☒ **Event free survival**<br>☐ **Toxicity**	☒ **Age-stratified analysis**<br>☐ **Comparison to other age-groups mentioned**
Centres:<br>Centers in Ontario (IMPACT Cohort is an Ontario population–based cohort)<br><br>Country:<br>Canada<br><br>Treatment era:<br>1992-2011	Inclusion criteria and cancer diagnosis:<br>15-21 year olds diagnosed with ALL<br><br>Age at diagnosis:<br>Median (range) yr<br>Pediatric centers: mean 16 +/- 1 year<br>Adult centers: mean 19 +/- 1 year<br>p<0.001<br><br>follow-up (if applicable):<br>not specified	Name of protocol<br>Most pediatric-inspired protocols based on DFCI Protocol 91-01<br><br>Patients treated in pediatric hospitals (123) received pediatric protocols<br><br>Patients treated in adult hospitals (55.3%, 152) received either pediatric (46; 30%) or adult protocols (106).	Definition of outcomes<br>- EFS, OS: from the time of initial diagnosis to relapse, progressive disease, death, or subsequent malignancy<br>- Induction death: within 28 days of diagnosis<br>- Treatment related mortality (TRM): any death occurring after diagnosis in the absence of another cancer event (relapse, progressive disease, subsequent malignancy)<br><br>Main results (for analysis)<br>- 5-year EFS, treated between 2006-2011: pediatric center AYA 80.8% ±5.8%, adult center AYA with ped. protocol 71.8% ±7.2%, adult centers with adult protocols 60.0% ±11.0%; p=0.02	Analysis<br>- chi squared tests or Fisher's exact tests for categorical variables<br>- t-test for continuous variables<br>- Kaplan-Meier approach with log rank test for EFS and OS<br>- univariate and multivariable Cox Proportional Hazards regression models for predictors of EFS and OS<br>- Competing risks analyses were for risk of TRM over time, cumulative incidence function approach for risk of TRM; these risks were compared by loci of care using Gray's test.<br>- Significance was $P < 0.05$.<br><br>Limitations:

			- 5-year OS, treated between 2006-2011: pediatric center AYA 90.9% ±4.3%, adult center AYA with ped. protocol 76.9% ±6.82%, adult centers with adult protocols 65.0%±10.7.0%; p=0.004<br><br>- 5-year EFS overall: pediatric center 74.2 ± 4.0 versus adult center 56.6±4.0; p=0.03<br>- 5-year EFS 1992-1998: pediatric center 64.5±8.6 versus adult center 50.9±6.9; p=0.16<br>- 5-year EFS 1999-2005: pediatric center 68.8±6.7 versus adult center 47.5±7.9; p=0.04<br>- 5-year EFS 2006-2011 : pediatric center 81.8±5.8 versus adult center 67.8±6.1; p=0.08<br>- 5-year OS overall pediatric center 82.1±3.5 versus adult center 63.8±3.9; p=0.0006<br>- 5-year OS 1992-1998 pediatric center 74.2±7.9 versus adult center 58.5±6.8; p=0.13<br>- 5-year OS 1999-2005 pediatric center 79.2±5.9 versus adult center 57.5±7.8; p=0.02<br>- 5-year OS 2006-2011 pediatric center 90.9±4.3 versus adult center 72.9±5.8; p=0.02<br>- Uni- and multivariate analysis for locus of care only and not the treatment protocol<br><br><u>Toxicity</u><br>- No difference in induction deaths between AYA at pediatric vs adult centers (<2% in both; *P* = 0.44).	- Unavailability of data about compliance with the protocol, dose reduction<br>- Retrospective, small sample size for some subgroups<br><br><u>Strength:</u><br>Chart abstraction was carried out with real-time validation by clinical experts<br><br><u>Other considerations:</u><br>Cytogenetics was comparable in both types of centers (disease biology likely not the cause of survival disparity), but the AYA treated in pediatric institutions were slightly younger<br><br>JBI Tool: Quality 1

			- 2-yr cum. incidence of TRM 5.6% +/- 2.1% for pediatric center AYA vs 5.9% +/- 1.9% for adult center AYA (*P* = 0.95)<br>- Restricted to last period pediatric vs adult 2-year TRM was 2.3% +/- 2.3% vs 3.4% +/-2.4% (*P* = 0.88).	

Hayakawa F, et al. Markedly improved outcomes and acceptable toxicity in adolescents and young adults with acute lymphoblastic leukemia following treatment with a pediatric protocol: a phase II study by the Japan Adult Leukemia Study Group
Blood Cancer Journal, 2014

**Study design**<br>**Treatment era**	**Participants**	**Treatment**	**Main outcomes**	**Additional remarks**
☐ **Clinical trial**<br>**Observational**<br>☒ **Cohort**<br>☐ **Cross-sectional**<br>☐ **Other:**<br>__________<br><br>☐ **Retrospective**<br>☒ **Prospective**	Study population (N)<br>➢ Original cohort: 150<br>➢ Eligible cohort: 139<br>➢ Analyzed cohort: 139		☒ **Overall survival**<br>☐ **Event free survival**<br>☒ **Toxicity**<br>**DFS**	☐ **Age-stratified analysis**<br>☒ **Comparison to other age-groups mentioned**
<u>Centres:</u><br>59 hospitals participating in the JALSG (Japan Adult Leukemia Study Group)<br><br><u>Country:</u><br>Japan<br><br><u>Treatment era:</u><br>August 2002 - October 2009	<u>Inclusion criteria and cancer diagnosis:</u><br>patients aged 15–24 years with BCR–ABL negative ALL<br><br><u>Age at diagnosis:</u><br>Median (range) yr<br>19 (15-24)<br><br><u>follow-up (if applicable):</u><br>Median (range)<br><br>ALL202-U 5.1 years<br>ALL97-U 5.8 years	<u>Name of protocol</u><br>Conventional adult protocol ALL97 versus pediatric- inspired ALL202<br><br>Results of Ph-negative ALL patients aged < 25 years in the JALSG ALL97 study (conventional adult protocol ALL97-U) used as reference.	<u>Definition of outcomes</u><br>- DFS: time from the date of achieving CR to relapse, death or the last visit<br>- OS as the time from the first day of therapy to death or the last visit.<br>- CR <5% blasts in bone marrow, no leukemic blasts in peripheral blood, recovery of peripheral blood values to neutrophil counts of at least 1.0 × 109/l and platelet counts of at least 100 × 109/l, and no evidence of extramedullary leukemia<br>- Toxicty according to National Cancer Institute Common Toxicity Criteria (NCI-CTC) Version 2.0<br><br><u>Main results (for analysis)</u><br>- CR rate for ALL202 94% (95% CI 88–97%) vs 84% (95% CI 75–90%) for ALL97<br>- 5-year DFS for ALL202 67% (95% CI 58–75%,) vs 44% ALL97-U	<u>Analysis</u><br>- Kaplan–Meier product limit method and to compare DFS and OS log-rank test<br>- Cox proportional hazard model for uni- and multivariate analyses.<br><br><u>Limitations:</u><br>Adherence to the protocol was low, mainly due to the high toxicity of this treatment. Protocol therapy was frequently terminated because of adverse events and the patients' wishes. Such therapy terminations were the most frequent during maintenance therapy, perhaps due to low motivation.<br><br><br>JBI Tool: Quality 1

			- 5-year OS for ALL202 73% (95% CI 64–80%) vs 45%<br>- DFS rate: for ALL202 71% versus ALL97-U 54% in standard-risk group<br>- DFS rate: for ALL202 63% versus ALL97-U 28% in high-risk group<br>-<br><u>Toxicity</u><br>Sepsis, hepatic toxicity and neuropathy were more frequent in ALL202-U, although no patient died from the adverse events associated with chemotherapy during post-remission therapy in this study.<br><br>The median delays from the planned schedule were 7 (range 0 to 171), 7 (range − 9 to 35), 9 (range 0 to 36), 6 (range – 8 to 70) and 19 (range − 5 to 62) days in induction, consolidation, sanctuary, reinduction and reconsolidation therapy, respectively.<br><br>L-asparaginase dose reductions were required for 48 (35%), 18 (18%) and 38 (47%) patients because of its adverse events in induction, reinduction and maintenance therapy, respectively.<br>Seventeen (30%) patients could complete the whole therapy without dose reductions in any drugs.<br>Fifty-seven (41%) patients could complete the whole therapy and 81 (59%) dropped out of the protocol therapy. Twenty-two (16%) patients terminated protocol therapy because of severe adverse events.	

Important points for manuscript/discussion etc:

**Hough R, et al.** Efficacy and toxicity of a paediatric protocol in teenagers and young adults with Philadelphia chromosome negative acute Lymphoblastic leukaemia: results from UKALL 2003<br>British Journal of Haematology, 2016				
**Study design<br>Treatment era**	**Participants**	**Treatment**	**Main outcomes**	**Additional remarks**
☒ **Clinical trial**<br>**Observational**<br>☐ **Cohort**<br>☐ **Cross-sectional**<br>☐ **Other:**<br>__________<br><br>☐ **Retrospective**<br>☒ **Prospective**	Study population (N)<br>➢ Original cohort: 3207<br>➢ Eligible cohort: 3126<br>➢ Analyzed cohort: 229	UK paediatric ALL<br>trial, UKALL2003<br><br>multi-centre, prospective,<br>randomized<br>phase III trial	☒ **Overall survival**<br>☒ **Event free survival**<br>☒ **Toxicity**	☒ **Age-stratified analysis**<br>☐ **Comparison to other age-groups mentioned**
Centres:<br>45 centers<br><br>Country:<br>UK, Ireland<br><br>Treatment era:<br>1 October 2003 - 30 June 2011	Inclusion criteria: Aged 16-24 years, diagnosed with Ph-negative B-ALL<br><br>Age at diagnosis:<br>Median (range) yr<br>18 (16-24)<br><br>follow-up:<br>Median (range) yr<br>5 years 10 months (range: 1 month – 10 years 1 month); at least 2-5 years for all TYA patients	Name of protocol<br>UKALL2003	Definition of outcomes<br>- EFS: time from diagnosis to relapse, secondary tumour or death,<br>- OS<br>- Cumulative risk of relapse<br>- Toxicity: National Cancer Institute Common Terminology Criteria for Adverse Events version 3.0<br><br>Main results (for analysis)<br><br>If age-stratified analyses<br>- Patients aged ≥16 years were more likely to be MRD high risk compared to younger patients<br>- Five-year EFS for entire population: 87.3% (95% CI: 86.1–88.5)<br>- Five-year EFS by age<br>- patients age under 10 years: 89.8% (88.4–91.2)<br>- 10 – 15 years: 83.6% (80.5–86.7)<br>- ≥16 years and 72.3% (66.2–78.4) OR = 2.1 (95% CI: 1.7–2.4), P (trend) < 0.00005, P(10–15 vs. ≥16) = 00004]<br>Age stratified analysis :	Analysis<br>- Chi Square tests<br>- Kaplan– Meier curves, compared them with the log-rank method<br>- All analyses by intention to treat.<br>- Two-sided p values and considered significant when <0.05<br><br>Strength: prospective study<br><br>JBI Tool: Quality 1

			- Five-year OS: for entire population: 91.6% (90.6–92.6)<br>- 5-year OS<br>- 16-24 years: 76.4% (70.5–82.3)<br>- 10-15 years: 87.5% (84.8–90.2)<br>- Under 10 years: 94.2% (93.2–95.2)<br>OR = 2.7 (2.2–3.4), P(trend) < 0.00005, P(10–15 vs. ≥16) = 0.0004]<br>- 5-year RR by age<br>- 16-24 years: 20.9% (15.0–26.8)<br>- 10-15 years: 10.7% (8.0–13.4)<br>- Under 10 years: 7.1% (5.9–8.3)<br>OR = 2.1 (1.7–2.6), P(trend) < 0.00005, P(10–15 vs. ≥16) = 0.0003]<br>- 5-year risk of death in remission (DIR):<br>- under 10 years: 2.1% (1.5–2.7)<br>- 10 -15 years: 3.4% (1.8–5.0)<br>- 16-24 years: 6.1% (2.8–9.4)<br>OR = 2.0 (1.4–3.9), P(trend) = 0.0007.<br>- incidence of SAEs was higher for > 10 or older compared < 10 years; OR (<10 years vs. 10– 24 years): 2.58 (95% CI: 2.24–2.95), P < 0.00005. Difference remained after stratifying for sex, NCI risk group, immunophenotype, MRD risk group and treatment allocation, adjusted OR 1.51 (95% CI: 1.28–1.77), P < 0.00005.<br>- The time to first SAE was significantly shorter and cumulative incidence of SAEs was significantly higher in those aged 10 years or older	

Valtis, et al. Orthopedic toxicities among adolescents and young adults treated in DFCI ALL Consortium Trials
Blood advances, 2022

**Study design**<br>**Treatment era**	**Participants**	**Treatment**	**Main outcomes**	**Additional remarks**
☐ **Clinical trial**<br>**Observational**<br>☒ **Cohort**<br>☐ **Cross-sectional**<br>☐ **Other:**<br>__________<br><br>☒ **Retrospective**<br>☐ **Prospective**	Study population (N)<br>➢ Analyzed cohort: 367	**Pediatric-type protocols (early and late-type)**	☐ **Overall survival**<br>☐ **Event free survival**<br>☒ **Toxicity**	☒ **Age-stratified analysis**<br>☐ **Comparison to other age-groups mentioned**
<u>Centres:</u><br>DFCI ALL Consortium and DFCI/Brigham and Women's Hospital, Massachusetts General Hospital, and Boston Children's Hospital<br><br><u>Country:</u><br>USA<br><br><u>Treatment era:</u><br>2000- 2018	<u>Inclusion criteria:</u><br>Participant of DFCI ALL Consortium protocols between 2000 and 2018 and aged >= 15 years at time of diagnosis. Patients aged >= 15 not enrolled on the studies but treated per the same protocols<br><br><u>Cancer diagnosis:</u><br>ALL<br><br><u>Age at diagnosis:</u><br>Age, median (range), years<br>Median 23 years (15- 50 years)<br>15-19 years: n=138 (38%)<br>20-29 years: n=110 (30%)<br>30-39 years: n=62 (17%)<br>40-50 years: n=57 (16%)<br><br><u>follow-up (if applicable):</u><br>Median 4.9 years (range, 0.08-14.1 years)	<u>Name of protocol</u><br>DFCI ALL Consortium protocols 00-001, 05-001, 01-175, 06-254<br><br>Early-generation protocols (00-001 and 01-175) accounted for 32% (n 5 117) of patients, with the remaining patients (68% [n 5 260]) treated on or as per late-generation protocols (05-001 and 06-254).	<u>Definition of outcomes</u><br>Symptomatic ostreonecrosis (ON) and fracture.<br><br>Events identified through chart review verified by magnetic resonance imaging, radiographs, or computed tomography imaging (selection of imaging modality for verification according to provider discretion).<br><br><u>Main results (for analysis)</u><br>- ON after late-generation peg-asparaginase-based protocols with 5-year cumulative incidence of 24% (95% CI, 18-30) versus early-generation native E. coli asparaginase-based protocols with 5-year cumulative incidence of 5% (95% CI, 2-10); HR, 5.28 (95% CI, 2.24-12.48); P= .001<br><br><u>Age-stratified analyses</u><br>- ON in patients <30 years had 5-year cumulative incidence of 21% (95% CI, 16-27) versus patients 30-50 years with 5-year cumulative incidence of 8% (95% CI, 4-14); univariate hazard ratio 2.77 (95% CI, 1.35- 5.65; P=.004)<br>ON 5-year cumulative incidence (29% CI)<br>15-19 years: 18 (12-25)<br>20-29 years: 25 (17-36)<br>30-39 years: 12 (5-23)	<u>Analysis</u><br>- 5-year cumulative incidences by Gray test<br>- Univariate and multivariable competing risk regression models with death as a competing risk.<br>- Multivariable models included age (<30 years vs >=30 years), sex, body mass index (BMI; underweight or normal vs overweight vs obese/morbidly obese), and treatment regimen backbone<br>- P values are two-sided and considered significant if < 0.05<br><br><u>Limitations:</u><br>- retrospective<br><br><br>Quality (JBI: Cohort study): Quality 1

			40-50 years: 4 (1-11)<br>p 0,003	

**Toft et al.** Results of NOPHO ALL2008 treatment for patients aged 1–45 years with acute lymphoblastic leukemia<br>Leukemia, 2018				
**Study design<br>Treatment era**	**Participants**	**Treatment**	**Main outcomes**	**Additional remarks**
☐ **Clinical trial**<br>**Observational**<br>☒ **Cohort**<br>☐ **Cross-sectional**<br>☐ **Other:** ____________<br><br>☐ **Retrospective**<br>☒ **Prospective**	Study population (N)<br>➢ Original cohort: 1591<br>➢ Analyzed cohort: 1509		☒ **Overall survival**<br>☒ **Event free survival**<br>☒ **Toxicity**	☒ **Age-stratified analysis**<br>☐ **Comparison to other age-groups mentioned**
Country:<br>Denmark, Estonia, Finland, Iceland, Lithuania, Norway and Sweden.<br><br>Treatment era:<br>July 2008 to December 2014	Inclusion criteria and cancer diagnosis:<br>patients aged 1–45 years with Philadelphia chromosome-negative B-cell precursor (BCP) or T-lineage ALL.<br>Exclusion of patients with Down's syndrome<br><br>Age at diagnosis:<br>1-9 years: 1022 (68%)<br>10-17 years: 266 (18%)<br>18-45 years: 221 (14%)<br><br>follow-up:<br>Median 4.6 years<br>(range: 3–6.4 years),	Name of protocol<br>NOPHO ALL2008	Definition of outcomes<br>- EFS = time from diagnosis until first occurrence of induction death, relapse, death in remission, development of a second malignancy, date of hSCT minus 2 weeks for the HR-hSCT patients or the last known follow-up for patients without events<br>- OS = time from diagnosis to death<br>- Death in complete remission (DRC1) = death without evidence of leukemia or a second malignancy.<br><br>Main results (for analysis)<br>5-years OS (HR, 95% CI)<br>1-9 years :0.94+/-0.01 (ref.)<br>10-17 years: 0.87 +/-0.02 (2.3; 1.5-3.5) p <0.001<br>18-45 years: 0.78+/-0.03 (3.8; 2.5- 5.7) p <0.0015-5-years EFS (HR, 95% CI)<br>1-9 years :0.89+/-0.01 (ref.)<br>10-17 years: 0.8 +/-0.03 (2.0; 1.4-2.8) p <0.001<br>18-45 years: 0.74+/-0.04 (2.8; 2.0- 4.0) p <0.001	Analysis<br>- χ^2 test or Fisher's exact test for categorical variables<br>- one-way ANOVA for continuous variables<br>- Spearman's correlation coefficient (rs) to identify possible associations for continuous variables.<br>- Kaplan–Meier to estimate probability of event-free survival (pEFS) and overall survival (pOS) rates, and differences were compared<br>with the 2-sided log-rank test.<br>- P-values were calculated using the Gray's test.<br>- All tests were two-sided with P<0.05 considered statistically significant.<br><br>Strength:<br>- intention to treat analysis<br>- prospective

			- risk of induction death did not differ significantly between the three age groups (P = 0.87).<br><br>- Toxicity: incidences of 19 toxicities were very similar for children and adults, except for the risk of thrombosis (P<0.001), pancreatitis (P<0.001) and osteonecrosis (P<0.001), which was higher for patients >10 years.<br>Risk of asparaginase-associated allergic reaction was significantly higher for<br>children below 10 years compared with older patients (P<0.001)<br><br>1-9 vs. 10-17 vs. 18-45 years<br>- ICU admission: 14.4% vs. 20.6% vs. 18.9% (p 0.5)<br>- peripheral paralysis: 9.9% vs. 11.5% vs.9.4% (p 0.45)<br>- anaphylactic reaction to ASP: 9.9% vs. 11.5% vs.9.4% (p 0.45)<br>- invasive fungal infection: 9.7% vs. 12.2% vs.13.2% (p 0.92)<br>- pancreatitis: 9.9% vs. 11.5% vs.9.4% (p 0.45)<br>- Hyperlipidemia: 7.1% vs. 9.9% vs.7.1% (p 0.12)<br>- Thrombosis: 3.6% vs. 15.3% vs.17.5% (p <0.001)<br>- ON: 2.3% vs. 13.4% vs.8.5% (p <0,001)<br>- Seizures: 3.8% vs. 6.1% vs.2.4% (p 0.88)<br>- PRES: 3.7% vs. 3.4% vs.2.4% (p 0.37)	Quality (JBI: Cohort study): Quality 1

Toft et al., Toxicity profile and treatment delays in NOPHO ALL2008—comparing adults and children with Philadelphia chromosome-negative acute lymphoblastic leukemia
European Journal of Haematology, 2016

Study design<br>Treatment era	Participants	Treatment	Main outcomes	Additional remarks
☐ **Clinical trial**<br>**Observational**<br>☒ **Cohort**<br>☐ **Cross-sectional**<br>☐ **Other:**<br>__________<br><br>☐ **Retrospective**<br>☒ **Prospective**	Study population (N)<br>➢ Original cohort: 1162 patients<br>➢ Eligible cohort: 1076<br>➢ Analyzed cohort: 1076		☐ **Overall survival**<br>☐ **Event free survival**<br>☒ **Toxicity**	☒ **Age-stratified analysis**<br>☐ **Comparison to other age-groups mentioned**
Country:<br>Sweden, Norway, Denmark, Finland, Iceland, Estonia, and Lithuania<br><br>Treatment era:<br>July 2008 to April 2013	Inclusion criteria and cancer diagnosis:<br>patients aged 1–45 years with Philadelphia chromosome-negative B-cell precursor (BCP) or T-lineage ALL. Patients with Down's syndrome were excluded<br><br>Age at diagnosis:<br><br>1–9.9 years: 69%, median 3 years<br>10–17.9 years: 18%, median 14 years<br>18–45 years: 13 %, median 26 years<br><br>Follow-up :<br>Median 3.3 years (1.03–5.93 yrs)	Name of protocol<br>NOPHO ALL2008 protocol.	Definition of outcomes<br>- SAE included 18 predefined events<br><br>Main results (for analysis)<br>- Increased duration of high-risk blocks as well as delayed intensification 1 and consolidation 2 for SR patients with increasing age group (p < 0.0001 and p 0.01 respectively).<br><br>Age stratified analysis:<br>% of toxic events during induction (from youngest to oldest age group):<br>- Heart failure 0-0-0-2.9-0, p 0.008<br>- Anaphylactic reaction 0.4-0-0-0-1.6, p 0.45<br>- ON 0-0.8-0-0-0, p 0.31<br>- Hyperglycemia 0.8-5.1-3.9-8.6-7.8, p <0.0001<br>- Abdominal catastrophe 0.8-1.7-0-1.4-0, p 0.52<br>- Liver dysfunction0.8-2.5-0-2.9-3.1, p 0.07<br>- VOD: 1-0-0-0-0, p 1.0<br>- Severe kidney dysfunction 0.4-1.7-1.3-1.4-0, p 0.17<br>- Bleeding 0.8-0-0-2.9-0, p 0.32<br>- Thrombosis 0.8-1.7-7.8-5.7-1.6, p <0.0001<br>- PRES 1-1.7-0-2.9-1.6, p 0.27<br>- Coma 0.4-0.8-0-0-0, p 0.77<br>- Seizures 1.2- 1.7-3.9-2.9-0, p 0.19<br>- Peripheral paralysis 2-1.7-1.3-2.9-6.2, p 0.26	Analysis<br>- Chi-square test or Fisher's exact test for categorical variables<br>- Spearman's correlation test to compare nonparametric or ordinal variables and analyzing for trends<br>- Bonferroni correction applied only when comparing durations of treatment phases to take account of the number of tests performed.<br>- multivariable logistic regression analysis if two or more variables were found to predict a toxicity or SAE<br>- All tests were two-sided with P < 0.05 considered statistically significant.<br><br>Strength: prospective<br><br>Other considerations:<br>- SAE vs no severe AE is not defined<br><br><br>Quality (JBI: Cohort study): Quality 1

			- Septic shock 1.6-2.5-1.3-0-0, p 0.69<br>- ICU admission 5.7-8.5-5.2-8.6-1.6, p 0.31<br>Hyperglycemia more common >9 years (overall p < 0.0001) and 18–28 yrs (OR = 11.3 (95% CI: (2.9;43.5); p 0.0002).<br>- Thrombosis more frequent in 15–17 years (OR 10.2 (2.6;39.1), p 0.0004) and 18–28 yrs (OR 7.3 (1,5;31.7), p 0.007)<br><br>- 49.8% had toxic events after induction<br>- incidence of at least one toxic event increased with increasing age group being 44.5%, 57.6%, 62.3%, 64.0%, and 64.2% for patients 1–9, 10–14, 15–17, 18–26, and 27–45 yrs (P < 0.0001)<br><br>% of toxic events (from youngest to oldest age group):<br>- Heart failure 0.7-0.8-0-2.6-1.3, p 0.29<br>- Anaphylactic reaction 12.7-11-6.5-10.7-3, p 0.09<br>- ON 01.5-11-6.5-5.3-6, p <0.0001<br>- Pancreatitis 6.1-6.8-9.1-13.3-9, p 0.16<br>- Hyperglycemia 3-6.8-5.2-6.7-4.5, p 0.11<br>- Abdominal catastrophe 1.2-1.7-2.6-2.7-1.5, p 0.47<br>- Liver dysfunction 3-4.2-2.6-2.7-4.5, p 0.83<br>- VOD: 2-3.4-0-2.7-0, p 0.38<br>- Severe kidney dysfunction 1.4-3.4-6.5-2.7-7.5, p 0.002<br>- Hypertension 1.6-2.5-0-0-0, p 0.51<br>- Thrombosis 3.5-9.3-16.9-16-16.4, p <0.0001<br>- PRES 2.7-4.2-1.3-4-0, p 0.41<br>- Coma 1.4-2.5-2.6-1.3-0, p 0.52<br>- Seizures 3.8- 5.1-6.5-1.3-3, p 0.5<br>- Peripheral paralysis 7.2-11.9-6.5-9.3-11.9, p 0.3<br>- Fungal infection 6.9-10.2-13-8-19.4, p 0.006<br>- PCP 3-3.4-6.5-5.3-6, p 0.24<br>- ICU admission 14.7-18.6-19.5-22.7-19.4, p 0.28<br>- Bleeding/CNS catastrophe 1.4-2.5-2.6-8-3, p 0.009	

			- Odds ratio for thrombosis 5.4 (2.6-11.0), 5.1 (2.4-10.4) and 5 (2.2-10.8) for patients 15–17, 18-26 and 27-45 years compared with children 1–9 years respectively (all $p < 0.0001$).<br>-Odds ratio for avascular osteonecrosis for patients 10–<br>14, 15-17, 18-26, 27-45 years 10.4 (4.4-24.9, $p < 0.0001$), 6.3 (1.9-18.3, p 0.001), 4.9 (1.3-15; p 0.009) and 6.6 (1.8-21.2, p 0.003) compared to 1-9 years respectively.	

Tantiworawit et al., Outcomes of adult acute lymphoblastic leukemia in the era of pediatric-inspired regimens: a single-center experience
International Journal of Hematology, 2019

**Study design**<br>**Treatment era**	**Participants**	**Treatment**	**Main outcomes**	**Additional remarks**
☐ **Clinical trial**<br>**Observational**<br>☒ **Cohort**<br>☐ **Cross-sectional**<br>☐ **Other:**<br>__________<br><br>☒ **Retrospective**<br>☐ **Prospective**	Study population (N)<br>➢ Analyzed cohort: 107<br><br>PIR 35 (33%)<br>Adult 75		☒ **Overall survival**<br>☐ **Event free survival**<br>☐ **Toxicity**	☐ **Age-stratified analysis**<br>☐ **Comparison to other age-groups mentioned**
<u>Centres:</u><br>Chiang Mai University (CMU) Hospital, Chiang Mai, Thailand<br><br><u>Country:</u><br>Thailand<br><br><u>Treatment era:</u><br>January 2007 to December 2017	<u>Inclusion criteria and cancer diagnosis:</u><br>Acute lymphoblastic leukemia patients, aged 15–65 years<br><br><u>Age at diagnosis:</u><br>Median 26 years (range 15–63 years)<br><br>Adult regimen: 29.5 years (range of 16–63)<br>Pediatric-inspired: 24 years (range of 15–39)<br><br><u>Follow-up:</u><br>Median 11.6 months (range 1–120).	<u>Name of protocol</u><br>modified Thai Pediatrics Oncology Group (TPOG)<br>vs<br>Hyper-CVAD or GMALL<br>Hyper-CVAD included tyrosine kinase inhibitors for patients with Ph+ ALL<br><br>67.3% received adult regimen (45 and 27 patients received Hyper-CVAD and GMALL, respectively))<br>32.7% (n=35) received pediatric-inspired regimen	<u>Definition of outcomes</u><br>- 2-year OS defined as the interval from time zero to death from any cause.<br>- Response rate, relapse rate, refractory rate, duration of continuous CR defined<br><br><u>Main results (for analysis)</u><br>- 2-year OS was 31.2% and 50.8% in the adult and pediatric groups (HR 1.52, 95% CI 0.83–2.78)<br>- Subgroup (n=86) of Philadelphia-negative ALL: 2-year DFS was 46.8% in pediatrics and 18.7% for adult treatment (HR 2.16, 95% CI 1.16–4.01). 2-year OS of 59.4% and 31.8% were shown for the pediatric-inspired and adult protocols (HR 2.03, 95% CI 1.04–3.96)<br><br>-CR rates adult vs pediatric protocol 79.2% and 88.2%, respectively ($P = 0.232$)<br>- relapse rate of 54.2% with adult regimen and 34.3% with pediatric-inspired regimen ($P < 0.01$).<br>- median DFS of 9 months and 19 months with 2-year DFS of 24.7% and 47.1% in the adult and pediatric groups, respectively, hazard ratio [HR], 1.73, 95% CI 1.22–3.03<br>-overall mortality adult vs. pediatric 68.1% and 40%; induction mortality not significantly different	<u>Analysis</u><br>- $\chi 2$ test for comparisons<br>- DFS and OS curves by Kaplan–Meier method<br>- comparison of outcomes in two treatment regimens by log-rank test<br>- calculation of adjusted hazard ratio (HR) by Cox proportional hazards regression model<br>- Statistical significance was defined as a two-tailed p value < 0.05.<br><br><u>Limitations:</u><br>No randomization<br>Median age of patients treated with adult protocol was significantly higher<br><br><u>Other considerations:</u><br>Some Ph+ patient didn't receive TKI<br><br>JBI Tool: Quality 2

Rytting et al. Augmented Berlin-Frankfurt-M€unster Therapy in Adolescents and Young Adults (AYAs) With Acute Lymphoblastic Leukemia (ALL)
Cancer 2014

**Study design**<br>**Treatment era**	**Participants**	**Treatment**	**Main outcomes**	**Additional remarks**
☒ **Clinical trial**<br>**Observational**<br>☒ **Cohort**<br>☐ **Cross-sectional**<br>☐ **Other:** ___________<br><br>☐ **Retrospective**<br>☒ **Prospective**	Study population (N)<br>➢ Analyzed cohort: 85<br>Historical cohort of 71 patients treated with Hyper CVAD		☒ **Overall survival**<br>☐ **Event free survival**<br>☒ **Toxicity**	☒ **Age-stratified analysis**<br>☐ **Comparison to other age-groups mentioned**
<u>Country:</u><br>USA<br><br><u>Treatment era:</u><br>October 2006 - April 2012<br><br>*Comparison with historic Hyper-CVAD cohort, treated November 2002 to September 2011	<u>Inclusion criteria:</u><br>Patients aged 12 – 40 years with Ph chromosome-negative ALL<br><br><u>Cancer diagnosis:</u><br>Ph chromosome-negative ALL;<br>pre-B-cell (n=69) and T-cell (n=16)<br><br><u>Age at diagnosis:</u><br>Median (range) 21 yrs [13-39]<br><br>*HyperCVAD cohort: 26 [16-40]<br><br><u>Follow-up:</u><br>Median follow-up of 40 months (range 4-75 months)	<u>Name of protocol</u><br>augmented Berlin-Frankfurt-M€unster (ABFM) regimen<br><br>*Historical cohort treated with Hyper-CVAD	<u>Definition of outcomes</u><br>- complete response (CR)= <5% bone marrow blasts and a normal peripheral blood count.<br>- Induction deaths= all deaths before day 29 of treatment<br>. CRD= complete remission duration<br>- Relapse= recurrence of ALL at any site<br>-Toxicities= according to the National Cancer Institute Common Toxicity Criteria, version 3.0.<br><br><u>Main results (for analysis)</u><br>ABFM versus hyper-CVAD:<br>- no statistically significant differences in CR rate (ABFM, 94% CR rate; hyper-CVAD, 99% CR rate, p 0.14)<br>- no statistically significant differences in CRD or OS rates between ABFM and hyper-CVAD<br>- 3-year OS rate: 74% with ABFM versus 71% with hyper-CVAD<br>- 3-year CRD rate: 70% with ABFM versus 66% with hyper-CVAD.<br>- OS and CRD analyzed in multivariate analyses (inlc. age, presenting WBC count, MRD status at the end of induction therapy): no statistically	<u>Analysis</u><br>- Differences in CR rates by chi-square tests or Fisher exact tests.<br>- Unadjusted CRD and OS analyses by Kaplan-Meier plots<br>- characteristics associated with differences in CRD and OS assessed using the log-rank test<br>- A P value <0.05 was considered statistically significant.<br><br><u>Limitations:</u><br>- Low power, thus no significant p values<br>-pediatric vs adult comparison done with a historic cohort<br><br><u>Strength:</u> prospective study<br><br>JBI Tool: Quality 1

			significant differences in outcome between the treatment regimens (data not shown)<br><br><u>Age stratified analysis</u><br>- The 3-year OS and CRD rates were 85% and 72%, respectively, for patients aged <21 years and 60% and 69%, respectively, for patients aged >21 years (P=0.055).<br>ty (≤21 years vs. >21 years):<br><br>- Allergic reaction to ASP: 20 vs.22%<br>- Grade 3 hypofibrinogenemia 10 vs. 21%, p 0.006<br>- Pancreatitis 9 vs. 12%<br>- Grade 3-4 elevated liver enzymes 27 vs. 44%<br>- Grade 3-4 elevated bilirubin 39 vs. 39%<br>- ON 14 vs. 7%<br>- Thrombosis 18 vs. 27%<br>- Stroke-like event 2 vs. 5%<br>- Grade 3-4 neuropathy 4 vs. 5%<br>None other than hypofibrinogenemia significant<br>- Grad 3-4 hypofibringogenemia more frequent in Patients > 21 years (P=.006)	

**Rytting et al.** Final results of a single institution experience with a pediatric-based regimen, the augmented Berlin–Frankfurt–M€unster, in adolescents and young adults with acute lymphoblastic leukemia, and comparison to the hyper-CVAD regimen<br>AmJHematology, 2016				
**Study design**<br>**Treatment era**	**Participants**	**Treatment**	**Main outcomes**	**Additional remarks**
☒ **Clinical trial**<br>**Observational**<br>☐ **Cohort**<br>☐ **Cross-sectional**<br>☐ **Other:**<br>__________<br><br>☐ **Retrospective**<br>☒ **Prospective**	Study population (N)<br>➢ Analyzed cohort:<br>106 in the pediatric arm and 102 in the adult regimen arm		☒ **Overall survival , CR, CRD**<br>☐ **Event free survival**<br>☒ **Toxicity**	☒ **Age-stratified analysis**<br>☐ **Comparison to other age-groups mentioned**
Centres:<br>University of Texas MD Anderson Cancer Center, Texas<br><br>Country: USA<br><br>Treatment era:<br>October 2006- March 2014	Inclusion criteria and cancer diagnosis:<br>AYA patients with Philadelphia chromosome-(Ph) negative ALL<br><br>Age at diagnosis:<br>Pediatric: Median 22 yr (13-39)<br>Adult: Median 27 yr (15-40)<br><br>Follow-up:<br>- median follow-up 66 months (range 17–107 months)<br>on ABFM<br>- median follow-up 88 months (range 1–152 months) on hyper-CVAD.	Name of protocol<br>Pediatric: Augmented Berlin–Frankfurt–Münster (ABFM) regimen<br><br>Adult: hyper CVAD (Hystorical cohort)	Definition of outcomes<br>CR= <5% blasts in the bone marrow and normal peripheral blood counts, in the absence of extramedullary disease<br>Induction death= deaths prior to Day 29 of treatment (Day 42 if extended induction)<br>Relapse= recurrence of ALL at any site<br>Toxicities= defined by National Cancer Institute Common Terminology Criteria, version 3.0.<br>Complete remission duration (CRD)was measured from the date of CR until relapse<br><br>Main results (for analysis)<br>CR 93% achieved CR on ABFM, and 98% on hyper-CVAD<br>5- year OS of 60% with ABFM and 60% with hyper-CVAD.<br>The 5-year CRD rates of 53% in ABFM and 55% in hyper-CVAD (p=0.98).<br><br>Age-stratified analyses<br>For patients ≤21 years, the 5-year OS rates were 65 and 68%, with ABFM and Hyper-CVAD respectively.	Analysis<br>- chi squared or Fisher's exact tests for differences in CR rates.<br>-<br>Kaplan–Meier method for CRD and OS.<br>- Characteristics associated with differences in CRD and OS assessed by log-rank testing.<br>- Cox proportional hazard regression was used to evaluate factors predicting CRD and OS<br>- P value of <0.05 was considered statistically significant.<br><br>Strength: prospective<br><br>JBI Tool: Quality 1

			For patients >21 years the 5-year OS rates were 57 and 58%, with ABFM and Hyper-CVAD respectively.<br><br>(the differences were not statistically significant).<br><br>Toxicty<br>With pediatric protocol more common hypofibrinogenemia, hyperbilirubinemia, pancreatitis. Infections in CR and during induction more common in Hyper CVAD;<br><br>Toxicty PIR vs adult<br>- Allergic reaction to ASP 19 vs. 11%, p 0.23<br>- Grade 3-4 hypofibrinogenemia 35 vs.14%, p <0.001<br>- Pancreatitis 11 vs. 3%, p 0.02<br>- Grade 3-4 liver enzymes increase 41 vs 44%, p 0.6<br>- Grade 3-4 bilirubin increase 38 vs. 18%, p 0.001<br>- ON 9 vs. 8%, p 0.68<br>- Thrombosis 19 vs. 12%, p 0.16<br>- Stroke-like event 3 vs. 0%, p 0.09<br>- Grade 3-4 neuropathy 6 vs. 4%, p 0.56<br>- Induction infections grade 3-4 22 vs. 45%, p <0.001<br>- Induction bleeding grade 3-4 1 vs. 5%, p 0.09<br>- Infections in CR in the first 60 days 30 vs. 60%, p <0.001<br>- Bleeding in CR in the first 60 days 1 vs. 5%, p 0.09<br>- Deaths in CR 8 vs. 7%, p, 0.85	

Ribera et al. Comparison of the Results of the Treatment of Adolescents and Young Adults With Standard-Risk Acute Lymphoblastic Leukemia With the Programa Espan˜ol de Tratamiento en Hematologı´a Pediatric-Based Protocol ALL-96
JOURNAL OF CLINICAL ONCOLOGY, 2008

**Study design**<br>**Treatment era**	**Participants**	**Treatment**	**Main outcomes**	**Additional remarks**
☒ **Clinical trial**<br>**Observational**<br>☒ **Cohort**<br>☐ **Cross-sectional**<br>☐ **Other:**<br>__________<br><br>☐ **Retrospective**<br>☒ **Prospective**	Study population (N)<br>➢ Original cohort: 331<br>➢ Eligible cohort: 81<br>➢ Analyzed cohort: 81 aged 15-30 years<br><br>Comparison between adolescents (n = 35) and young adults (n = 46)		☒ **Overall survival**<br>☒ **Event free survival**<br>☒ **Toxicity**	☒ **Age-stratified analysis**<br>☐ **Comparison to other age-groups mentioned**
<u>Country:</u><br>Spain<br><br><u>Treatment era:</u><br>June 1996- June 2005	<u>Inclusion criteria and cancer diagnosis:</u><br>Standard risk ALL, adolescents (15 to 18 years) and young adults (19 to 30 years)<br><br><u>Age at diagnosis:</u><br>20 years (range, 15 to 30 years)<br><br><u>follow-up:</u><br>Median follow-up: 4.2 years (range, 2 to 10 years).	<u>Name of protocol</u><br>PETHEMA ALL-96 protocol<br>Pediatric-inspired	<u>Definition of outcomes</u><br>- CR= absence of clinical manifestations of ALL<br>- EFS= time from diagnosis to failure, relapse, death or last follow-up.<br>- Overall survival (OS)= time from study entry to death or last follow-up.<br><br><u>Main results (for analysis)</u><br>- OS rate at 6 years: 69% (95% CI, 59% to 81%)<br>- 6-year EFS 61%, (95% CI, 51% to 72%)<br>- No significance between adolescents (15-18 years) and young adults (19-30 years) in EFS (60% and 95% CI, 43% to 77% *v* 63% and 95% CI, 48% to 78%; *P*=.97; Fig 1) or OS (77% and 95% CI, 63% to 91% *v* 63% and 95% CI, 46% to 80%; *P* = .44)<br><br><u>Toxicity</u><br>There was a higher number of grade 1 infections in young adults (13 young adults *v* one adolescent; *P*=.007).<br>Grade 4 neutropenia: 44% in adolescents (median duration 5 days, range, 1-14 days) vs 59% of young adults (median duration, 5 days; range, 1-17 days)	<u>Analysis</u><br>- t-test, Mann-WhitneyUtest to compare quantitative variables<br>- chi squared or Fisher's exact test to assess differences in proportions.<br><br>Kaplan- Meier method for EFS and OS and compared by the log-rank test.<br><br><u>Limitations:</u><br>Not enough power<br><br><u>Strength:</u> prospective<br><br>JBI Tool: Grade 1

			Grade 4 thrombocytopenia: 10% if adolescents (median duration 5 days, range, 4 to 7 days) vs. 33% in of young adults ((median duration, 3 days; range, 1-14 days).<br>Delays during reinductions significantly more frequent in young adults than in adolescents (median duration of maintenance-1.7 and 6.4 months, respectively; *P*=.04).<br>modifications of asparaginase or vincristine were performed in 19% of cycles in adolescents *vs. 33%* young adults; *P* 0.03	

**Ribera et al.** A pediatric regimen for adolescents and young adults with Philadelphia chromosome-negative acute lymphoblastic leukemia: Results of the ALLRE08 PETHEMA trial<br>Cancer Medicine 2020				
**Study design<br>Treatment era**	**Participants**	**Treatment**	**Main outcomes**	**Additional remarks**
☒ **Clinical trial**<br>**Observational**<br>☒ **Cohort**<br>☐ **Cross-sectional**<br>☐ **Other:**<br>__________<br><br>☐ **Retrospective**<br>☒ **Prospective**	Study population (N)<br>➢ Original cohort: 102<br>➢ Eligible cohort: 89<br>➢ Analyzed cohort: 89<br><br>Adolescents (*n* = 38)<br>Young adults (*n* = 51)		☒ **Overall survival**<br>☒ **Event free survival**<br>☒ **Toxicity**	☒ **Age-stratified analysis**<br>☐ **Comparison to other age-groups mentioned**
Centres:<br><br>Country:<br>Spain<br><br>Treatment era:<br>August 2008- April 2018	Inclusion criteria and cancer diagnosis:<br>Adolescents (15 to 18 years) and young adults (19 to 30 years) with standard-risk (SR) Ph-neg B-cell precursor ALL or T-ALL<br><br><br>Age at diagnosis:<br>Median 20 yeas (range: 15-29)<br>Adolescents: 17 years (15-18)<br>Young adults: 23 years (19-23)<br><br>Follow-up:<br>median 4.19 years (range 0.04-<br>9.47)<br>Time since diagnosis (if applicable)<br>Median (range) yr	Name of protocol<br>PETHEMA ALLRE08<br>Pediatric inspired	Definition of outcomes<br>Primary endpoints: i) CR rate, ii) cumulative incidence of relapse (CIR), and iii) overall survival (OS).<br>Overall survival= time from study entry to death or last follow-up.<br>Event-free survival= time from diagnosis to failure, relapse or death by any cause or last follow-up.<br>The Common Terminology Criteria for Adverse Events (CTCAE v 3.0) used for analysis of toxicity.<br><br>Main results (for analysis)<br>5 years OS: in adolescents 87% (95%CI: 74%-100%) vs 63% (46%-80%) in young adults (P = 0.021)<br>5-year EFS: in adolescents 78% (95%CI 59-89) vs 49% (95%CI 31-65%) in young adults (p=0.151)<br>5-year CIR: In adolescents 13% (96%CI 4%-28%) vs 52% (34%-67%) in young adults (p = 0.012)<br><br>Toxicity not reported age stratified<br>There were no differences between adolescents and YA in drug modifications and delays, and these modifications did not show impact on patients' outcome.	Analysis<br>- median test to compare quantitative variables<br>- Chi-square or Fisher's exact tests to assess differences in proportions<br>- Kaplan Meier methods for OS and EFS and compared by the log-rank test.<br><br>Limitations:<br>Univariate analysis only, low power<br><br>Strength: prospective<br><br><br>JBI Tool: Quality 1-2

Quist-Paulsen et al., T-cell acute lymphoblastic leukemia in patients 1–45 years treated with the pediatric NOPHO ALL2008 protocol
Leukemia, 2019

**Study design**<br>**Treatment era**	**Participants**	**Treatment**	**Main outcomes**	**Additional remarks**
☐ **Clinical trial**<br>**Observational**<br>☒ **Cohort**<br>☐ **Cross-sectional**<br>☐ **Other:**<br>__________<br><br>☐ **Retrospective**<br>☒ **Prospective**	Study population (N)<br>➢ Original cohort: 1815<br>➢ Analyzed cohort: 278		☒ **Overall survival**<br>☒ **Event free survival**<br>☐ **Toxicity**	☒ **Age-stratified analysis**<br>☐ **Comparison to other age-groups mentioned**
Centres:<br>NOPHO group<br><br>Country:<br>NOPHO countries<br><br>Treatment era:<br>July 2008 to March 2016, follow-up for events until March 2019	Inclusion criteria and cancer diagnosis:<br>T-ALL in the age group 1–45<br><br>Age at diagnosis:<br>Age 18-45 n=83<br>Age 10-17 n=78<br>Age 1-9 n=117<br><br>Age 18-45 median 27 years<br>Age 10-17 median 14 years<br>Age 1-9 median 5 years<br><br>follow-up (if applicable):<br>median follow-up time of 5.9 years<br>1-9 age group 6.3 yrs (4.0–8.3)<br>10-17 age group 5.9 (4.4–7.6)<br>18-45 age group 5.7 (4.7–6.8)	Name of protocol<br>NOPHO ALL2008 protocol	Definition of outcomes<br>Events = induction deaths, death in first complete remission (DCR1), relapse, resistant disease, and second malignancy.<br><br>Main results (for analysis)<br>- Cumulative risk (95%CI) for relapse: age 1-9=13.9 (8.7-21.7).; age 10-17= 8.1 (3.7-17.2), age 18-45= 21.4 (13.8-32.2)<br>- Cumulative risk (95%CI) for death in first remission: age 1-9=4.3 (1.8-10.1), age 10-17=9.2 (4.5-18.4), age 18-45=12.3 (6.8-21.6)<br>- Further calculated but not relevant for this review: cumulative risk (95%CI) for death from any cause, all events<br>- 5-year overall survival (95%CI): age 1-9=0.82 (0.74-0.88; ref.), age 10-17=0.76 (0.66-0.86; p=0.3), age 18-45=0.65 (0.55-0.75; p=0.01)<br>→OS significantly higher in the 1–9 years group compared to adults. The 10–17 years group and adults did not differ significantly<br><br>- 5-year event-free survival (95%CI): age 1-9=0.80 (0.72–0.88), age 10-17=0.75 (0.65–0.85), age 18-45=0.64 (95% CI 0.52–0.76)<br><br>Toxicity not reported for diff. age groups.	Analysis<br>- Differences in patient characteristics by $\chi 2$ tests for categorical variables and Mann–Whitney U tests (if two groups) or Kruskal–Wallis tests (if three groups) for continuous variables<br>- Kaplan–Meier method and log-rank test for differences in overall and event-free survival.<br>- Life tables with the Wilcoxon test were used to estimate, and compare between groups, 5-year overall and event-free survival.<br>- Cox regression for event-specific hazard ratios according to age groups,<br>- two sided tests with significance level below 0.05.<br><br>Strength: prospective<br><br>JBI Tool: Quality 1

Kliman et al. Comparison of a pediatric-inspired treatment protocol versus standard-intensity chemotherapy for young adults with standard-risk BCR-ABL negative acute lymphoblastic leukemia
Leukemia & Lymphoma, 2017

**Study design<br>Treatment era**	**Participants**	**Treatment**	**Main outcomes**	**Additional remarks**
☐ **Clinical trial**<br>**Observational**<br>☐ **Cohort**<br>☒ **Cross-sectional**<br>☐ **Other:**<br>__________<br><br>☒ **Retrospective**<br>☐ **Prospective**	Study population (N)<br>➢ Analyzed cohort: 47<br><br>22 pediatric protocol<br>25 adult protocol		☒ **Overall survival**<br>☒ **Event free survival**<br>☒ **Toxicity**	☐ **Age-stratified analysis**<br>☐ **Comparison to other age-groups mentioned**
Centres:<br>General Hospital, University of British Columbia, Vancouver<br><br>Country:<br>Canada<br><br>Treatment era:<br>pediatric protocol: 02/2008 -11/2014<br>adult protocol: 02/2003- 07/2008	Inclusion criteria and Cancer diagnosis:<br>Patients with standard- risk BCR-ABL negative acute lymphoblastic leukemia aged 18–40 years<br><br>Age at diagnosis:<br>Median 24.5 years (range 18-40)<br>Pediatric protocol: median 27.6 years<br>Adult protocol: median 23.5 years<br><br>follow-up: Combined: median 40.1 months;<br>Pediatric protocol: median 36.8 months<br>Adult protocol: median 73.1 months	Name of protocol<br>Pediatric regimen: modification of the pediatric-inspired protocol 01-175 of the Dana Farber Cancer Institute<br><br>Comparative standard adult ALL protocol: lower cumulative doses of agents than pediatric protocol, no HDMTX and no HiDAC	Definition of outcomes<br>- Treatment related toxicity or death related to therapy included up to 30 days after completion of therapy<br>-Toxicity = infection necessitating inpatient admission, invasive fungal infection and pancreatitis or thrombotic events due to asparaginase.<br>-Overall survival = calculated from time of diagnosis until death or last follow up<br>- Event-free survival = calculated from diagnosis until death, failure to achieve remission on post-induction bone marrow biopsy, relapse, or last follow up for patients who are alive and in continuous CR<br><br>Main results (for analysis)<br>- CR after induction: 100% in pediatric protocol vs 86% in adult protocol (p=0.095)<br>- Relapse: pediatric protocol 28% vs adult protocol 45% (p=0.214)	Analysis<br>- Estimation of OS and EFS by Kaplan–Meier method<br>Patient characteristics compared using Chi squared test or Fisher's exact test for binary variables and Mann–Whitney test for continuous variables.<br><br>Limitations: low power, restrospective, not randomised<br><br>JBI Tool: Quality 2

			- 3-year OS: pediatric protocol 80% vs adult protocol 59%<br>- 3-year OS after censoring patients at time of transplant: pediatric protocol 86% vs adult protocol 68%<br>3-year EFS: pediatric protocol 80% vs adult protocol 45% (p=.019)<br><br>Toxicty<br>- Candidemia: pediatric protocol 8% vs adult protocol 9% (p=0.108)<br>- Severe infection: pediatric protocol 44% vs adult protocol 41% (p=0.831)<br>- Thrombosis: pediatric protocol 32% vs adult protocol 9% (p=0.079)<br>- Pancreatitis: pediatric protocol 8% vs adult protocol 0% (p=0.491)<br>- Death from toxicityy: pediatric protocol 0% vs adult protocol 5% (p=0.468)<br>No differences in toxicities between protocols were observed (no data shown).	

**Rank et al.** Thromboembolism in acute lymphoblastic leukemia: results of NOPHO ALL2008 protocol treatment in patients aged 1 to 45 years<br>Blood, 2018				
**Study design<br>Treatment era**	**Participants**	**Treatment**	**Main outcomes**	**Additional remarks**
☐ **Clinical trial**<br>**Observational**<br>☒ **Cohort**<br>☐ **Cross-sectional**<br>☐ **Other:** ________<br><br>☐ **Retrospective**<br>☒ **Prospective**	Study population (N)<br>➢ Original cohort: 1861<br>➢ Analyzed cohort: 1772<br><br>1-9.9 years: 1192 (67%)<br>10-17.9 years: 306 (17%)<br>18-45 years: 274 (16%)		☐ **Overall survival**<br>☐ **Event free survival**<br>☒ **Toxicity**	☒ **Age-stratified analysis**<br>☐ **Comparison to other age-groups mentioned**
Centres:<br>NOPHO centers<br><br>Country:<br>Denmark, Estonia, Finland, Iceland, Lithuania, Norway, and Sweden.<br><br>Treatment era:<br>July 2008- February 2016	Inclusion criteria and cancer diagnosis:<br>BCR-ABL negative ALL patients aged 1 to 45 years<br><br>Age at diagnosis:<br>Range 1-45<br><br>follow-up:<br>median follow-up of 4.3 years (interquartile range [IQR], 2.5-6.4 years)	Name of protocol<br>NOPHO ALL2008 (pediatric)	Definition of outcomes<br>Symptomatic/asymptomatic and venous/arterial TE cases identified through clinical evaluation and confirmed by imaging were included in this study and evaluated. Cases of superficial thrombophlebitis, septic embolism, and CVL dysfunction resulting from thrombosis without other symptoms were excluded<br><br>Main results (for analysis)<br>2.5-year cumulative incidence of any TE<br>1-9.9 years: 3.7% (2.64- 4.8)<br>10- 17.9 years: 15.5% (11.3- 19.4)<br>18- 45 years: 18.1% (13.2- 22.8)<br>p< .0001<br><br>Multivariate analysis with delayed entry at day 29 (HRa for TE, 95% CI)<br>1-9.9 years: ref.<br>10- 17.9 years: 4.9, 3.1-7.8, p <0.0001<br>18- 45 years: 6.06, 3.65-10.1, p <0.0001	Analysis<br>The median follow-up time estimated with reversed Kaplan-Meier method. The cumulative incidences of first TE estimated using the Aalen-Johansen estimator, considering relapse, death, and second malignant neoplasm as competing events, and the estimates were compared with Gray's test. Time to first TE was analyzed in a Cox proportional hazards regression model including relevant selected clinical-, disease-, and treatment-related characteristics.<br><br>Strength: prospective study with big cohort<br><br>Other considerations:<br>No common recommendations for routine antithrombotic prophylaxis exist in the ALL2008 protocol. There was no active screening for TE.<br><br>JBI Tool: Quality 1

			- The adjusted TE-specific hazard was significantly increased in patients aged 6.0 to 14.9 years (HRa, 2.0; 95% CI, 1.2-3.5; P5.01), 15.0 to 20.9 years (HRa, 7.74; 95% CI, 4.52-13.2; P , .0001), and 21.0 to 45.9 years (HRa, 6.54; 95% CI, 3.69-11.6; P , .0001), using 1.0 to 5.9 years as reference.<br><br>Patients aged 18.0- 45.9: increased hazard of PE compared with children younger than 10.0 years (HRa, 11.6, 95% CI: 4.02-33.7; p < 0.0001).<br><br>- Adolescents aged 10.0 to 17.9 years: increased hazard of CSVT compared with children younger than 10.0 years (HRa 3.3, 95% CI: 1.5-7.3; p 0.003)<br><br>- When analyzing time to death, the hazard of death was significantly increased in younger patients with TE compared with younger patients without TE ages 1.0 to 9.9 years (HRa, 10.1; 95% CI, 4.05-25.3; P < .0001) and 10.0 to 17.9 years (HRa, 4.51; 95% CI, 1.39-14.7; P 5 .01). No difference in hazard of death was seen when comparing patients aged at least 18 years with or without TE (HRa, 1.1; 95% CI, 0.2-5.0; P 5 .9).<br><br>Asparaginase was truncated in 38/128 patients with thromboembolism, whereas thromboembolism diagnosis was unassociated with increased hazard of relapse (P 5 .6).	
